# DHCR24 Deficiency Drives Age-Related Meibomian Gland Dysfunction by Regulating Lipid Metabolic Imbalance and Cytosolic mtDNA-Induced cGAS-STING Activation

**DOI:** 10.7150/ijbs.129636

**Published:** 2026-03-28

**Authors:** Yuchen Cai, Tianyi Zhou, Jiaming Sun, Xueyao Cai, Wenjun Shi, Junzhao Chen, Christos C. Zouboulis, Hao Sun, Yao Fu, Liangbo Chen

**Affiliations:** 1 Department of Ophthalmology, Shanghai Ninth People's Hospital, Shanghai Jiao Tong University School of Medicine, Shanghai, China.; 2 Shanghai Key Laboratory of Orbital Diseases and Ocular Oncology, Shanghai, China.; 3 Department of Plastic and Reconstructive Surgery, Shanghai Ninth People's Hospital, Shanghai Jiao Tong University School of Medicine, Shanghai, China.; 4 Departments of Dermatology, Venereology, Allergology and Immunology, Dessau Medical Center, Brandenburg Medical School Theodor Fontane and Faculty of Health Sciences Brandenburg, Dessau, Germany.

**Keywords:** DHCR24, aging, lipid dysregulation, meibomian glands, cGAS-STING, mtDNA

## Abstract

Dysregulated lipid metabolism and chronic inflammation are hallmarks of aging, yet their interplay in age-related tissue disorders remains poorly defined. In the ocular surface, age-related meibomian gland dysfunction (ARMGD) is highly prevalent but mechanistically unclear, leading to significant visual impairment without targeted therapies. To identify key molecular drivers of ARMGD, we performed integrated multi-omics screening of aging mouse meibomian glands (MGs) and identified DHCR24, a key cholesterol metabolism enzyme, as a critical regulator of gland homeostasis. Single-cell sequencing identified age-associated downregulation of *Dhcr24* predominantly in meibocytes. Based on this finding, we generated a meibocyte-specific *Dhcr24* knockout (cKO) model, which exhibited typical ARMGD pathology including glandular atrophy, disrupted lipid homeostasis, and inflammatory activation. Further *in vitro* studies using SZ95 sebocytes demonstrated that DHCR24 deficiency induces mitochondrial dysfunction and cytosolic mitochondrial DNA (mtDNA) leakage, triggering cGAS/STING-dependent inflammatory senescence. Notably, AAV-mediated restoration of DHCR24 in mice reversed age-related gland pathology. Our findings establish DHCR24 as a dual-target regulator that maintains cholesterol metabolic homeostasis while suppressing mtDNA-driven inflammation via the cGAS-STING pathway, highlighting its therapeutic potential for ARMGD and related disorders characterized by lipid-inflammatory imbalance.

## Introduction

Aging represents one of the most significant health challenges of the 21st century, driven by a progressive decline in cellular function and tissue homeostasis 1. This process is characterized by typical features, including genomic instability, mitochondrial dysfunction, metabolic abnormalities, and chronic low-grade inflammation, which contribute to the pathogenesis of age-related diseases 2,3. Among these, ocular conditions such as age-related macular degeneration (AMD), cataracts, and dry eye disease have emerged as major causes of visual impairment 4. In particular, meibomian gland dysfunction (MGD) has gained increasing recognition as a highly prevalent yet mechanistically elusive disorder 5. With aging being the primary risk factor for this condition, age-related MGD (ARMGD) now affects approximately 70% of the population aged over 60 years in the US, highlighting its clinical and public health significance 6,7.

ARMGD is a chronic and progressive condition characterized by abnormal lipid secretion and inflammation in the meibomian glands (MGs), significantly impacting patients' vision and quality of life 7. As specialized sebaceous glands of the eyelid margin, MGs provide essential lipids via holocrine secretion to the tear film, which are crucial for preventing tear evaporation and maintaining ocular surface homeostasis 8. Recent studies have identified significant age-dependent alterations in the lipid profiles of MGs, including imbalanced wax ester (WE) to cholesteryl ester (ChE) ratios, reduced phospholipid levels, and altered fatty acid profiles 9,10. In parallel, aged MGs exhibit increased inflammation, marked by elevated proinflammatory cytokine release and nuclear factor-κB (NF-κB) activation, which drives acinar atrophy and gland dropout 11. Current treatment modalities for ARMGD, such as warm compresses, eyelid hygiene, artificial tears, and anti-inflammatory agents, provide only symptomatic relief and fail to address the underlying pathogenic mechanisms 12,13. While advanced therapies such as thermal pulsation and intense pulsed light (IPL) have shown some efficacy in improving meibum delivery, they cannot reverse glandular atrophy or restore normal meibum composition 14. However, the intricate nature of the association between age-related lipid dysregulation and inflammatory activation in MGs remains poorly understood, posing a challenge for targeted therapeutic development.

The interplay between inflammation and lipid metabolism is increasingly recognized as a hallmark of aging across multiple organ systems. In conditions such as atherosclerosis, Alzheimer's disease, and metabolic syndrome, age-dependent activation of innate immune pathways, including the cyclic GMP-AMP synthase-stimulator of interferon genes (cGAS-STING) axis and NF-κB signaling, has been shown to disrupt lipid homeostasis, leading to cytotoxic lipid species accumulation, organelle stress, and tissue dysfunction 15-17. Pro-inflammatory cytokines such as interleukin-6 (IL-6) and tumor necrosis factor-α (TNF-α) not only amplify local inflammatory responses but also interfere with lipid synthesis and trafficking, resulting in altered lipid composition and impaired cellular function 18,19. Central to this immunometabolic interplay are regulatory factors such as peroxisome proliferator activated receptors (PPARs), whose age-related decline compromises both lipid metabolism and inflammatory resolution 20. Emerging evidence further underscores the vital role of cholesterol homeostasis in maintaining mitochondrial integrity. As a key component of mitochondrial membranes, cholesterol regulates membrane fluidity, permeability, and cristae architecture. Dysregulation of cholesterol metabolism can therefore compromise mitochondrial structure and function, potentially leading to the leakage of pro-inflammatory mitochondrial components, including mitochondrial DNA (mtDNA) 21-23. Notably, mitochondrial dysfunction and subsequent mtDNA release have been identified as critical triggers of cGAS-STING activation in age-related diseases, establishing a link between metabolic stress and inflammation 24,25. While these mechanisms are increasingly well-characterized in systemic and neurological disorders, their roles in ocular aging, particularly in ARMGD, remain largely unexplored. Given that MGs are highly active lipid-secreting organs that exhibit both inflammatory infiltration and lipid abnormalities with aging, it is plausible that similar immunometabolic crosstalk underlies their age-related decline. Therefore, a systematic investigation into how lipid dysregulation and inflammatory signaling impact MG function in ARMGD holds promise for identifying novel disease mechanisms for therapeutic intervention.

In this study, we employed integrative multi-omics screening of aging MGs and identified 24-dehydrocholesterol reductase (DHCR24), a key enzyme in cholesterol biosynthesis, as a pivotal regulator of ARMGD. To functionally characterize its role, we constructed a meibocyte-specific *Dhcr24* conditional knockout (cKO) mouse model using a meibocyte-enriched promoter identified from our single-cell sequencing data. Genetic ablation of *Dhcr24* recapitulated ARMGD pathology, including disrupted acinar architecture, aberrant lipid metabolism, and marked inflammatory activation. Mechanistically, DHCR24 deficiency induced mitochondrial dysfunction and cytosolic mtDNA leakage, leading to activation of the cGAS-STING signaling and subsequent NF-κB-mediated inflammation. Strikingly, AAV-mediated restoration of DHCR24 in mice effectively reversed age-related glandular pathology. Together, these results establish DHCR24 as a critical node linking cholesterol homeostasis, mitochondrial integrity, and inflammatory signaling in ARMGD, offering a mechanistic foundation for targeted therapies to restore MG function and potentially mitigate related age-associated disorders.

## Materials and Methods

### Animals models

Two-month and two-year-old female C57/BL6J mice were purchased from the Cyagen Biosciences Inc., China. We used female mice to maintain homogeneity in our model and to avoid potential confounding factors associated with male aggression or fluctuations in androgen levels. Ocular surface injury was induced using 0.2% benzalkonium chloride (BAC) eye drops, administered twice daily for 14 days 26. *Dhcr24^fl/fl^* mice (Strain S-CKO-16104; Cyagen Biosciences) were engineered via the CRISPR/Cas9 system, which introduced two loxP sites flanking exon 3. *Krt14-Cre^ERT^* mice (C001297; Cyagen Biosciences) were crossed with *Dhcr24^fl/fl^* mice to produce *Krt14-Cre^ERT^ Dhcr24^fl/fl^* (conditional knockout, cKO; *Dhcr24*-cKO) offspring, lacking *Dhcr24* expression in *K14* expressing meibocytes, with *Dhcr24^fl/fl^* mice serving as controls. Gene deletion was induced by administering tamoxifen (TAM) intraperitoneally at 20 mg/kg/day for 7 days.

All mice were maintained in pathogen-free environment and provided with a standard chow diet while subjected to a 12-hour light/dark cycle. Animals were randomly allocated to various experimental groups, ensuring that each cage housed mice from different groups. The procedures were conducted in accordance with the guidelines established by the Association for Research in Vision and Ophthalmology (ARVO) regarding the ethical use of animals in ophthalmic and vision research. Approval for all animal experiments was obtained from the Animal Ethics Committee of Shanghai Ninth People's Hospital, Shanghai Jiao Tong University School of Medicine (SH9H-2024-A1387-1).

### Immunohistochemistry (IHC) of patient samples

Human tarsal plate specimens were surgically obtained from a 3-year-old with congenital eyelid deformity and a 40-year-old with traumatic injury and were fixed in 4% paraformaldehyde. Informed consent was obtained from all patients or their legal representatives, and the study received ethical approval from the Institutional Review Board of Shanghai Ninth People's Hospital, Shanghai Jiao Tong University School of Medicine (SH9H-2021-T171-1).

Paraffin-embedded human tarsal plates were sectioned at 4 µm thickness and deparaffinized. Antigen retrieval was performed using a 10 mM citrate buffer at 95°C for 20 minutes. Sections were treated with 3% hydrogen peroxide to block endogenous peroxidase activity and were incubated overnight at 4 °C with anti-DHCR24 (Bioss; #bs-5390R). An HRP-conjugated secondary antibody was then applied, followed by DAB for visualization. Images were processed using ImageJ (IHC Profiler plugin). The H-score was calculated using the formula: H-score = (% of cells at low positive × 1) + (% at positive × 2) + (% at high positive × 3).

### Mouse ocular surface and MG clinical examinations

#### Corneal opacity and fluorescein test

A single ophthalmologist performed all examinations using a slit-lamp microscope to image mouse eyelid margins and corneas. Corneal opacity severity was evaluated following the Draize method, which involves multiplying the observed degree of opacity by the affected corneal area 9. To assess corneal epithelial integrity, 1 µL of a 1% sodium fluorescein solution (Jingming, China) was applied to the conjunctival sac. After 1 minute, fluorescein staining was observed and photographed under cobalt blue light. Staining intensity was scored according to the following criteria: 0 (no staining), 0.5 (slight punctate staining), 1 (widespread punctate staining), 2 (staining covering less than one-third of the cornea), 3 (staining covering more than one-third), or 4 (staining covering more than two-thirds) 27.

#### Rose Bengal staining

To evaluate subtle corneal epithelial damage, 5 μL of a 0.25% Rose Bengal solution (Aladdin, China) was applied into the conjunctival sac, followed by gentle blinking to distribute the dye. After rinsing with 0.9% saline, residual staining was photographed under a slit-lamp microscope. Staining intensity was scored per quadrant: 0 (no staining), 1 (punctate staining), 2 (punctate staining covering < 50%), 3 (non-confluent staining covering > 50%), or 4 (confluent staining). Total scores were calculated as the sum of all quadrants.

#### Tear secretion test

Tear production was assessed using the non-anesthetic Schirmer I test 28. Initially, excess tears were absorbed, and a phenol red cotton thread (Jingming, China) was placed in the lateral conjunctival sac for one minute. The wetted length (mm) was recorded three times per eye, and the average was calculated for analysis. Mice were gently held to minimize blinking during the measurements.

#### MG dissection and scoring

Following euthanasia, the upper and lower tarsal plates of the mice were dissected bilaterally and rinsed in phosphate-buffered saline (PBS). Under a microscope, micro-scissors were used to carefully separate the skin, subcutaneous tissue, and conjunctiva of the eyelids to obtain a pure sample of mouse MG tissue. MG morphology was photographed using a Keratograph (Oculus, Germany) and clinically scored as follows: 0 points for no irregularities or absence of MG; 1 point for irregular MGs without absence; 2 points for irregularities with less than one-third absence; and 3 points for irregularities with one-third or more absence. MG scores were assessed based on the photographs and presented as mean ± SD.

### Intra-MG adeno-associated virus (AAV) injection

Recombinant adeno-associated virus rAAV2/8 designed for *Dhcr24* overexpression was purchased from Genomeditech, China. Under anesthesia, 5 μL of rAAV2/8-DHCR24 (5 × 10^12^ VG/mL) or control AAV-vehicle was microinjected into the mouse MGs using a 34-gauge needle attached to a 10 μL microsyringe (Hamilton, Switzerland) under microscope. Two injections were performed at different points within each gland to ensure broader coverage. To analyze AAV tropism, mice were microinjected AAV-DHCR24 or saline solution, and MG sections were evaluated via direct fluorescence imaging two weeks post-injection.

### Histological examinations

Mouse eyelid tissues were paraffin-embedded or cryopreserved in tissue-freezing medium. Sagittal sections (6 μm thick) were prepared from both tissue types. Paraffin-embedded sections were subjected to H&E staining. Cryopreserved eyelid sections underwent Oil Red O (ORO) analysis. Briefly, sections were fixed in 4% paraformaldehyde (15 min), rinsed with PBS, incubated with fresh ORO solution (Servicebio, China) for 15 min, counterstained with hematoxylin (2 min), and finally mounted using an aqueous medium. To evaluate potential systemic effects, major organs including skin, heart, liver, spleen, lung, and kidney were collected, paraffin-embedded, sectioned, and stained with H&E.

### Transmission electron microscopy (TEM)

Freshly dissected MG tissues were immersed in 2.5% glutaraldehyde/0.1 M phosphate buffer (pH 7.4) for 24 hours at 4°C, followed by post-fixation with 1% osmium tetroxide for 2 hours. The samples were then dehydrated through a graded series of ethanol and embedded in Epon 812 epoxy resin. Ultrathin sections, measuring 70 nm, were cut using a Leica UC7 ultramicrotome, placed onto copper grids, and contrasted with uranyl acetate and lead citrate. Imaging was examined on a Hitachi HT7800 TEM.

### Multi-omics analyses of mouse MGs

#### Transcriptomic profiling

Total RNA was isolated from mouse MGs using the RNeasy Micro Kit (Qiagen), with integrity verified on an Agilent 2100 Bioanalyzer. Sequencing libraries were constructed with the TruSeq Stranded mRNA Library Prep Kit (Illumina) and sequenced on an Illumina NovaSeq 6000 platform (Shanghai Newcore Biotech). The raw sequencing reads were mapped to the mouse reference genome (GRCm38) and subsequently analyzed using edgeR (v3.28.1) and limma (v3.42.2) in R (v4.0.2). Differentially expressed genes (DEGs) were identified using thresholds of |fold change (FC)| > 1.2 and *p* < 0.05. Functional enrichment analyses of Gene Ontology (GO) and Kyoto Encyclopedia of Genes and Genomes (KEGG) were performed using clusterProfiler (v4.0.5). Principal component analysis (PCA), volcano plots, and heatmaps were visualized with ggplot2 (v3.3.5). The correlation between *Dhcr24* expression and lipid metabolism was assessed via Mantel test and Pearson correlation coefficient using the R package ggcor (v0.9.6).

#### Lipidomic profiling

Lipids were isolated from MG tissues using the methyl tert-butyl ether (MTBE) method. Briefly, tissue homogenization was performed in 400 μL ice-cold methanol spiked with internal standards, mixed with MTBE and centrifuged (14,000 × g, 15 min, 10°C). Lipid separation was achieved using an UltiMate 3000 UHPLC system (Thermo Fisher). The mobile phase gradient was optimized for effective lipid separation. Lipids were analyzed in both positive and negative ionization modes on a Q-Exactive Plus mass spectrometer (Thermo Fisher) by Shanghai Applied Protein Technology. LipidSearch (v4.2.3) software was utilized for lipid identification and quantification. Variable importance in projection (VIP) scores and orthogonal partial least squares discriminant analysis (OPLS-DA) and were calculated using MetaX (v1.6.0). Lipids meeting VIP > 1 and* p* < 0.05 were classified as differentially expressed. Lipid saturation was quantified via LipidSearch's fragmentation pattern analysis. GO, KEGG, and Gene Set Enrichment Analysis (GSEA) were performed for functional annotations. Sankey diagrams were created using the R package ggalluvial (v0.12.5), integrating lipid differential expression results with KEGG enrichment outcomes.

#### Proteomic profiling

MG tissues were lysed in RIPA buffer (Beyotime, China) containing PMSF, followed by ultrasonication. Protein concentration was quantified via the BCA assay. Proteins were subjected to tryptic digestion, further analyzed by liquid chromatography-tandem mass spectrometry (LC-MS/MS) using a Thermo Scientific Orbitrap mass spectrometer (analysis performed by Shanghai OE Biotech). For TMT labeling, samples were resuspended in TEAB, mixed with acetonitrile, and incubated. Labeled peptides underwent reverse-phase separation via HPLC and were processed on a Q-Exactive mass spectrometer. Raw MS data were analyzed via Proteome Discoverer (v2.4). Search parameters included trypsin digestion, carbamidomethylation (static), and oxidation (M)/acetylation (N-term) (dynamic). Protein quantification required ≥ 2 unique peptides and a false discovery rate (FDR) < 1%. Proteins exhibiting |fold change (FC)| > 1.2 and *p* < 0.05 were designated as differentially expressed proteins (DEPs). PCA and functional enrichment (GO/KEGG) were performed as described for transcriptomics.

### Single-cell RNA sequencing

Freshly dissected MGs from young (Y; 2-month-old, *n* = 1) and aged (O; 2-year-old, *n* = 1) mice were minced and digested. Dissociated cells were harvested and treated with Red Blood Cell Lysis Solution (Thermo Fisher) to remove erythrocytes. Cell viability (>85%) was confirmed via Trypan Blue staining using an automated cell counter. Single-cell suspensions were partitioned into Gel Beads-in-Emulsion (GEMs) using the Chromium Controller (10× Genomics) and Chromium Next GEM Single Cell 3′ Reagent Kits v3.1. cDNA synthesis and amplification were performed according to the manufacturer's protocol. Amplified cDNA libraries were quantified using a Bioanalyzer 2100 (Agilent) and Qubit 4.0 Fluorometer (Thermo Fisher). Paired-end sequencing (2 × 150 bp) was carried out on an Illumina NovaSeq 6000 platform. Raw sequencing reads (FASTQ files) were aligned to the mouse reference genome (mm10) using the STAR aligner. Raw data were processed and filtered using Cell Ranger v7.1.0 (10× Genomics) to remove empty droplets (criteria: nGene > 200, nUMI > 500, mitochondrial gene ratio < 20%). Unsupervised clustering based on transcriptional similarity partitioned cells into distinct subpopulations. Cluster-specific DEGs were identified via Seurat's FindAllMarkers. Dimensionality reduction was achieved through t-distributed stochastic neighbor embedding (t-SNE) and uniform manifold approximation and projection (UMAP) methods. Cell clusters were annotated using characteristic marker genes for each subpopulation, cross-referenced with the Cell Taxonomy database and established literature 29,30. Expression levels of *Krt14* and *Dhcr24* in meibocyte were extracted, and Pearson correlation coefficients were calculated using ggpubr (v0.6.0) in R.

### Cell culture and RNA interference

Immortalized human SZ95 sebaceous gland cells were maintained in DMEM (Gibco) containing 10% FBS (Gibco), 5 ng/mL recombinant human EGF (Peprotech), and 100 U/mL penicillin/streptomycin (Gibco) at 37 °C/5% CO_2_ 31. The medium was replaced every other day. For *DHCR24* knockdown, SZ95 sebocytes were transfected with 50 nM DHCR24-targeting siRNA (Genomeditech). Nontargeting siRNA (siNC) was used as control. The siDHCR24 sequence was as follows: 5′-CGCUUAUCUUCGAUAUCUAdTdT-3′ (forward), 5′-UAGAUAUCGAAGAUAAGCGdTdT-3′ (reverse). For the treatment of STING inhibitor H-151 (Targetmol, USA; #T5674), SZ95 cells were cultured with 1 µM H-151 for 12 h.

### Immunofluorescence (IF)

Mouse MG cryosections were permeabilized (0.1% Triton X-100), blocked with 5% BSA, and incubated with primary antibodies against KRT14 (Bioss; #bsm-52054R), DHCR24 (Bioss; #bs-5390R), IL-6 (Proteintech; 21865-1-AP), IL-17 (Bioss; #bs-1183R), TNF-α (Proteintech; #17590-1-AP), NF-κB p65 (Abcam; #ab32536), phospho-NF-kB p65 (S536) (Abcam; #ab76302), and STING (Proteintech; #19851-1-AP) overnight. Alexa Fluor 488/594 secondary antibodies (Invitrogen) were applied for 1 hour, and nuclei were counterstained with DAPI. Images were acquired using a Nikon A1R confocal microscope, and fluorescence intensity was analyzed with ImageJ software.

For mitochondrial double-stranded DNA (dsDNA) detection, siRNA-transfected SZ95 cells were fixed and permeabilized by utilizing 0.1% Triton X-100 for 20 min. Following the blocking of non-specific binding with 2% BSA, the cells were incubated with Tom20 (Proteintech; #66777-1-Ig) and dsDNA (Progen; #AC-30-10) antibodies overnight at 4 °C. After applying an Alexa Fluor 488/594-conjugated secondary antibody for 1 hour, immunofluorescence images were captured using a Leica Stellaris 5 confocal microscope.

### Quantitative reverse transcription-polymerase chain reaction (RT-qPCR)

Total RNA was extracted from MGs or cells using RNA extraction kit (TianGen). Subsequently, cDNA synthesis was performed with the PrimeScript RT Kit (Takara). For quantitative PCR, the ABI Prism 7000 (Applied Biosystems) was utilized, employing Hieff UNICON SYBR Green master mix (Yeasen) and conducting technical triplicates for accuracy. Normalization was achieved using the 2-ΔΔCt method relative to GAPDH, with primers sourced from Sangon Biotech (see Table S1).

### Cytosolic fraction and mitochondrial DNA extraction

Cytosolic fraction was performed as previously described 32. Briefly, SZ95 cells were harvested and centrifuged at 900g for 5 minutes. The supernatant was removed, and the cells were resuspended in PBS before being split into two aliquots of equal volume. After a second centrifugation at 600g for 5 minutes, the pellet from one tube was designated as the whole-cell fraction for subsequent measurement of nuclear DNA (nDNA) and mtDNA. The pellet from the other tube was incubated in 500 μL of buffer containing 25 mg/mL digitonin, 150 mM NaCl, and 50 mM HEPES (pH 7.4) for 10 minutes at room temperature. Following this, the cells were centrifuged at 150g at 4 °C. The supernatant was then centrifuged twice more at 150g at 4 °C and once at 17,000g for 10 minutes to obtain the cytosolic fraction. DNA Extraction was performed using DNA isolation kit (Tiangen) according to the maufacturer's instructions. DNA concentration was measured using the Nanodrop ND-1000 Spectrophotometer. qPCR was conducted on 10 ng of DNA using the SYBR qPCR master mix (Yeasen) on the ABI Prism 7000 instrument. The qPCR targets included mtDNA regions ND1 and DLOOP, as well as nDNA regions 18S and TERT. The cytosolic mtDNA to whole-cell nDNA ratio 33 and the cytosolic mtDNA to whole-cell mtDNA ratio were calculated 32,34.

### Enzyme-linked immunosorbent assay (ELISA)

MG lysates were used to assess the levels of murine IL-6, IL-17, and TNF-α using commercial ELISA kits (ELK Biotechnology, USA; #ELK1157 for IL-6, #ELK1147 for IL-17, and #ELK1387 for TNF-α) following the manufacturer's instructions. Absorbance was measured with a microplate reader (ELX800, BioTek), and protein concentrations were calculated from a standard curve.

### Western blot (WB)

Total tissue or cellular protein was extracted using RIPA lysis, followed by quantification using a BCA kit (Beyotime). The samples were then denatured by adding loading buffer and applying heat treatment. Electrophoresis was performed using sodium dodecyl sulfate-polyacrylamide gel electrophoresis (SDS-PAGE), after which the proteins were transferred to a PVDF membrane (Millipore). Immunodetection involved blocking with 5% skim milk, followed by incubation with primary antibodies at 4℃ overnight. Primary antibodies included DHCR24 (Proteintech; #10471-1-AP), NF-κB p65 (Abcam; #ab32536), phospho-NF-kB p65 (S536) (Abcam; #ab76302), cGAS (Proteintech; #26416-1-AP), STING (Proteintech; #19851-1-AP), phospho-STING (Ser366) (CST; #85735), GAPDH (ABclonal; #AC002), and β-Actin (ABclonal; #AC004). Afterward, HRP-conjugated secondary antibodies were applied at room temperature for 1 h. Visualization was achieved using an ECL chemiluminescence (Beyotime), and images were captured with the ChemiDoc System (Bio-Rad).

### Statistical analysis

Statistical analysis was performed using GraphPad Prism 7.0 and R version 4.0.4, with data presented as mean ± standard deviation (SD). Comparisons between two groups were conducted using Student's t-test, while comparisons among three or more groups utilized one-way analysis of variance (ANOVA). To account for multiple testing, the Benjamini-Hochberg method was applied. Significance thresholds were set at *p* < 0.05 (*); *p* < 0.01 (**); *p* < 0.001 (***), *p* < 0.0001 (****).

## Results

### Multi-omics profiling identified DHCR24 as a central regulator in ARMGD

**Figure 1:**
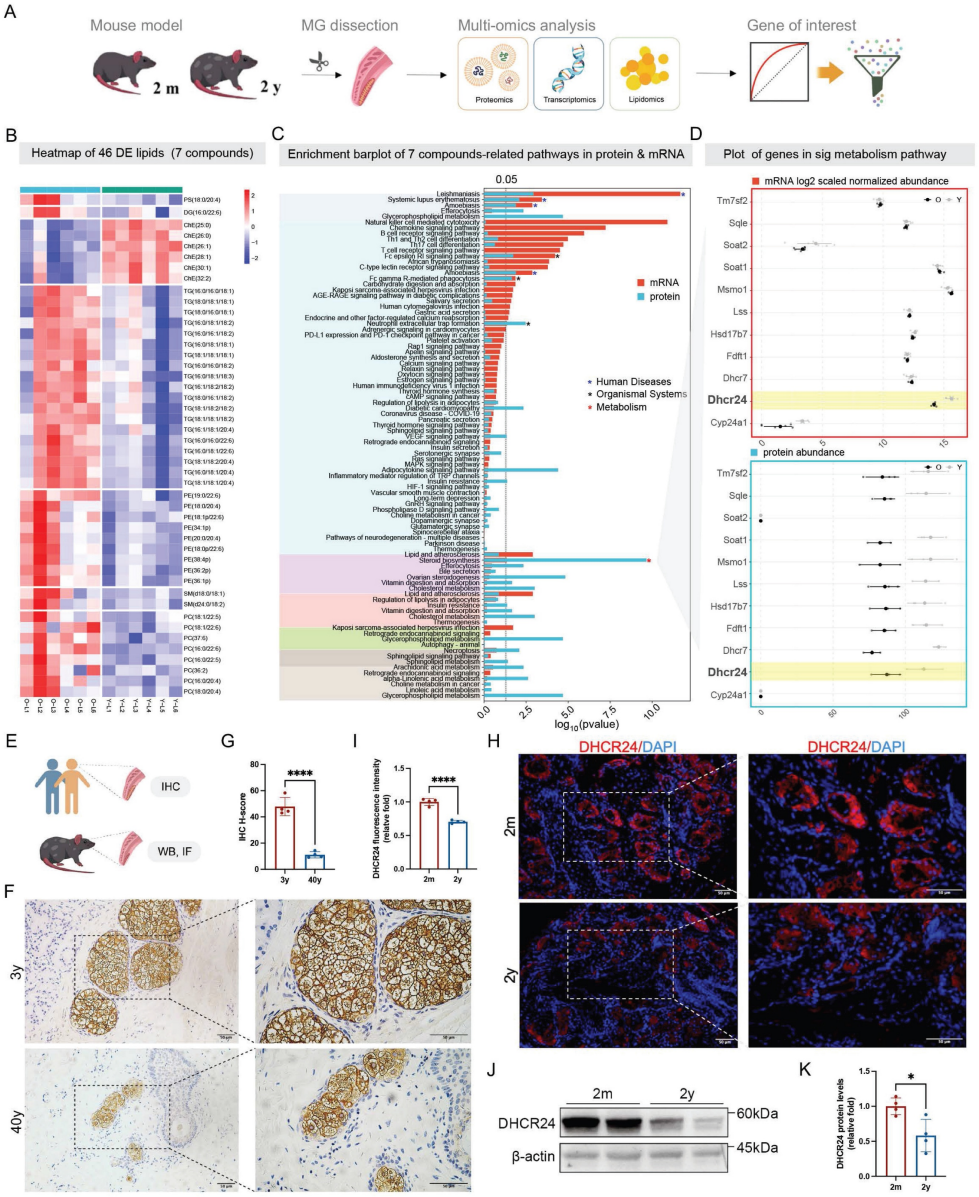
**Multi-omics analyses identified dehydrocholesterol reductase 24 (DHCR24) as a biomarker of age-related meibomian gland dysfunction (ARMGD).** (**A**) Multi-omics study design for biomarker screening. In general, meibomian glands (MGs) of two-month-old (*n* = 15) and two-year-old (*n* = 15) mice were dissected for lipidomics (6 vs. 6), proteomics (3 vs. 3), and transcriptomics (6 vs. 6) profiling, followed by systematic bioinformatics analyses for biomarker identification. (**B**) Heatmap visualization of 46 differentially expressed lipids in 7 classes (PE, SM, PS, PC, DG, ChE, and TG) between the two-month-old (Y) and two-year-old (O) groups. ChE, cholesteryl esters; DG, diglyceride; PE, phosphatidylethanolamine; PC, phosphatidylcholine; PS, phosphatidylserine; SM, sphingomyelin; TG, triglyceride. (**C**) Integrated proteomics and transcriptomics analyses identified the shared significant pathways (*p < 0.05; dashed line) in three categories, including human diseases, organismal systems, and metabolism. (**D**) Combined transcriptomics and proteomics analyses of steroid biosynthesis pathway identified DHCR24 as the only biomarker differentially expressed in both mRNA and protein levels between the two-month-old and two-year-old groups. (**E**) Schematic diagram for subsequent DHCR24 verification, including immunohistochemistry (IHC) for paraffin-embedded human tarsal plate samples, and Western blot (WB) and immunofluorescence (IF) for mouse MGs. (**F**,** G**) IHC staining and H-score for DHCR24 in 3-year-old and 40-year-old human tarsal plate samples. Scale bars: 50 μm. (**H**, **I**) IF and relative fluorescence intensity for DHCR24 in two-month-old and two-year-old mouse MGs. Scale bars: 50 μm. (**J**, **K**) DHCR24 protein expression in two-month-old and two-year-old mouse MGs.

To systematically identify molecular drivers of ARMGD, we conducted an integrated multi-omics analysis of MGs from young (2-month-old) and aged (2-year-old) mice. MGs from each age group (*n* = 15 per group) were subjected to lipidomic (6 vs. 6), proteomic (3 vs. 3), and transcriptomic (6 vs. 6) profiling, followed by systematic bioinformatics analysis (Fig. 1A). Before molecular analysis, ocular morphological examinations revealed age-related morphological degeneration characterized by glandular atrophy, ductal dropout, elevated MG pathology scores (Fig. S1A-C), and a significant reduction in lipid content as shown by ORO staining (Fig. S1D), consistent with clinical ARMGD features. Multi-omics datasets revealed clear molecular separation between young and aged MG samples (Fig. S2A-C). Lipidomic identified 46 significantly altered lipid species across major classes, including ChE, triglycerides (TG), sphingomyelin (SM), diglycerides (DG), and phospholipids (phosphatidylcholine [PC], phosphatidylethanolamine [PE], phosphatidylserine [PS]) (Fig. 1B). To visually integrate these findings, a Sankey diagram illustrates the flow from these dysregulated lipids to significantly enriched metabolic and inflammatory pathways, including cholesterol metabolism, glycerophospholipid metabolism, sphingolipid signaling, adipocytokine signaling, the NF-κB signaling pathway, autophagy, and efferocytosis (Fig. S3).

Moreover, integrated pathway analysis of transcriptomic and proteomic data highlighted significant dysregulation in immune-inflammation networks, such as chemokine signaling, T cell receptor signaling pathway, alongside lipid metabolic pathways including cholesterol metabolism, steroid biosynthesis, and glycerophospholipid metabolism (Fig. 1C). Notably, ChE levels were markedly reduced in aged mice (Fig. S4A), and pathway enrichment analyses highlighted disruptions in cholesterol metabolism and steroid biosynthesis at both the proteomic and transcriptomic levels (Fig. 1C and Fig. S4A). Strikingly, DHCR24, a key enzyme in cholesterol and steroid biosynthesis, emerged as the only gene/protein consistently downregulated at both the mRNA and protein levels in aged MGs (Fig. 1D), underscoring its potential role as a key regulator of age-related lipid dysfunction. In addition, the analysis of downregulated lipid classes, including DG, PC, PE, PS, SM, and TG, revealed their association with enriched metabolic and inflammatory signaling, such as glycerophospholipid metabolism, chemokine signaling pathway, necroptosis, and cAMP signaling pathway, which were further supported by transcriptomic and proteomic evidence (Fig. S4B).

Validation studies in human and murine tarsal plates confirmed age-dependent decline in DHCR24 (Fig. 1E). IHC of human samples revealed significantly lower DHCR24 levels in MGs from a 40-year-old donor compared to a 3-year-old child, as quantified by H-score (Fig. 1F, G). Consistent with this, IF and WB analyses in mice demonstrated a pronounced reduction of DHCR24 in aged (two-year-old) MGs relative to young (two-month-old) controls, evidenced by decreased fluorescence intensity (Fig. 1H, I) and diminished protein expression (Fig. 1J, K). Collectively, these results suggest DHCR24 as a pivotal factor in MG homeostasis during aging, with its loss linked to MGD pathogenesis.

### Single-cell sequencing indicated meibocyte-specific *Dhcr24* downregulation in aging MGs and the construction of meibocyte-specific *Dhcr24*-cKO mouse model

**Figure 2:**
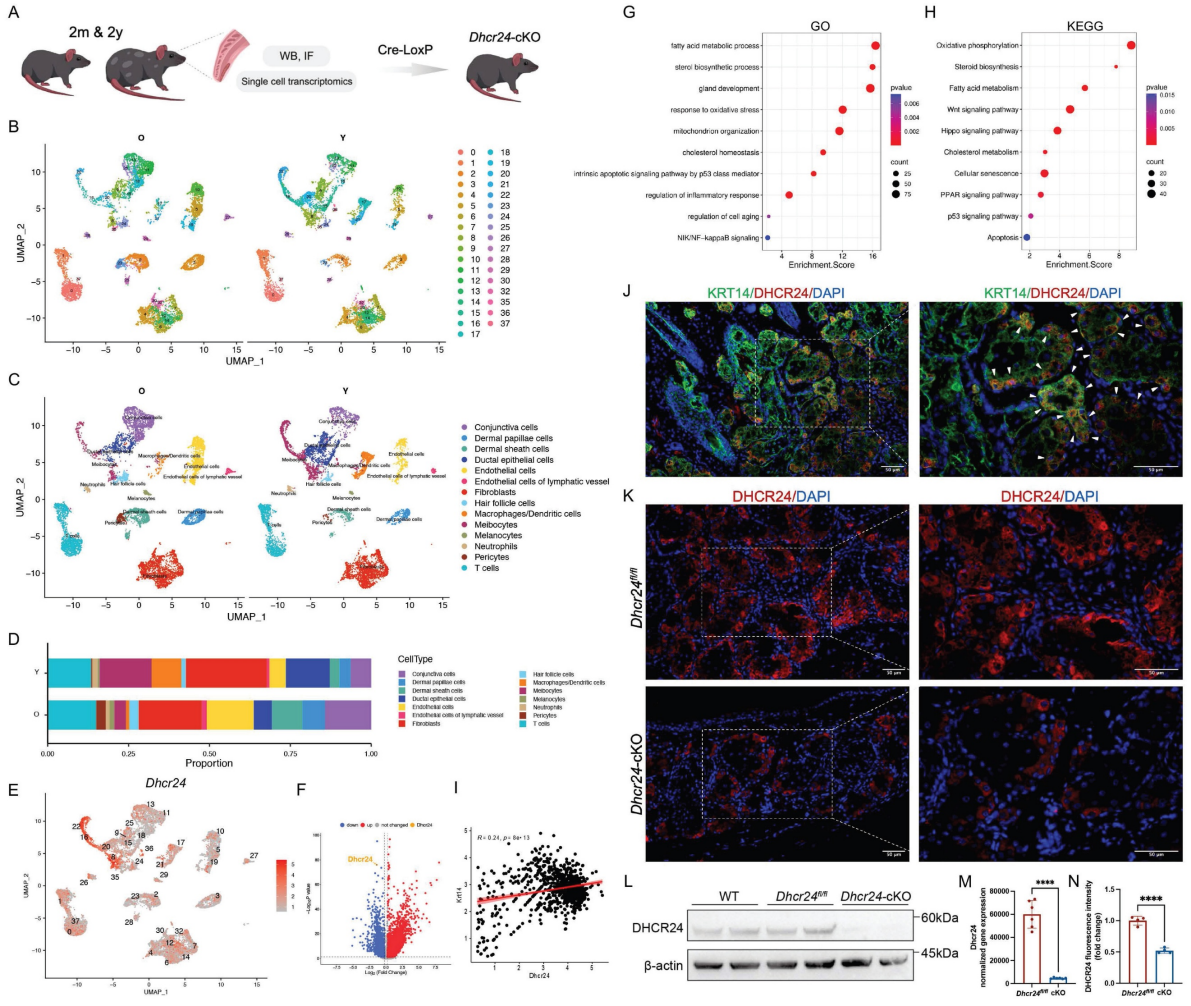
** Construction and validation of *Dhcr24* conditional knockout (cKO) mice.** (**A**) Study design of the single-cell RNA sequencing (scRNA-seq) of mouse MGs and construction of meibocyte-specific cKO mice. In brief, meibomian glands (MGs) of two-month (*n* = 1) and two-year-old (*n* = 1) female mice were dissected for scRNA-seq to identify the *Dhcr24*-expressing cells, followed by the construction of *Dhcr24*-cKO mice based on Cre-LoxP system. (**B**, **C**) Uniform manifold approximation and projection (UMAP) of young (Y) and aged (O) mouse MGs (**B**) classified MG cells into 37 distinct clusters, (**C**) annotated into 14 major cell types, with *Dhcr24* predominantly expressed in meibocytes. (**D**) Cell proportions for 14 cell types in MGs. (**E**) Scatter plot of *Dhcr24* expression in each cell cluster. (**F**) Volcano plot of differentially expressed genes (DEGs) in meibocytes comparing O vs. Y mice, showing significantly reduced *Dhcr24* expression. (**G**, **H**) Functional annotation of (**G**) Gene ontology (GO) and (**H**) Kyoto encyclopedia of genes and genomes (KEGG) for the DEGs in the meibocyte populations. (**I**) Pearson correlation analysis for the correlation between *Dhcr24* and *Krt14* expression in meibocyte. (**J**) Immunofluorescence (IF) staining confirmed co-localization (white arrows) of DHCR24 and KRT14 in two-month-old MG sections. Scale bars: 50 μm. (**K**) IF and (**N**) relative fluorescence intensity for DHCR24 in *Dhcr24*-cKO and *Dhcr24^fl/fl^* mouse MGs. Scale bars: 50 μm. (**L**) Western blot comparing DHCR24 expression in MGs of *Dhcr24*-cKO mice with wild type (WT) and *Dhcr24^fl/fl^* mice. (**M**) Normalized *Dhcr24* gene expression in *Dhcr24*-cKO vs. *Dhcr24^fl/fl^* MGs from transcriptome analysis.

To elucidate the cellular origins and functional implications of DHCR24, we performed single-cell RNA sequencing (scRNA-seq) on MGs from young and aged mice and generated meibocyte-specific *Dhcr24*-cKO mice using the Cre-LoxP system (Fig. 2A). Dimensional reduction of the scRNA-seq data was performed using PCA (Fig. S5A). UMAP and t-SNE analysis identified 37 distinct cellular clusters (Fig. 2B, Fig. S5B), further annotated into 14 subpopulations according to distinct marker genes (Fig. S5C), including meibocytes, ductal epithelial cells, conjunctival cells, dermal papillae and sheath cells, endothelial cells (including lymphatic subtypes), immune cells (macrophages/dendritic cells, T cells, neutrophils), fibroblasts, melanocytes, pericytes, and hair follicle cells (Fig. 2C and Fig. S5D). Notably, meibocytes, the lipid-secreting epithelial population critical for MG function, were markedly reduced in aged MGs, while conjunctival cells, T cells, and melanocytes exhibited age-dependent increase (Fig. 2D). *Dhcr24* expression was highly enriched in meibocyte-associated clusters (clusters 8, 16, and 22; Fig. 2E) but significantly downregulated in meibocytes of aged mice (Fig. 2F and Fig. S5E), indicating its essential role in meibocyte function during aging. Further functional analysis revealed significant dysregulation of multiple pathways in aged meibocytes, including cholesterol homeostasis, sterol biosynthesis, gland development, response to oxidative stress, mitochondrial organization, regulation of inflammatory responses, cellular aging, and key pathways such as NIK/NF-κB, PPAR, Wnt, and Hippo signaling pathways (Fig. 2G, H). These findings highlight the complex molecular landscape affecting meibocyte function and MG health in aging.

Keratin 14 (KRT14), a structural component of intermediate filaments essential for ocular surface integrity, is broadly expressed during MG development and critical for gland homeostasis 35. Further UMAP visualization showed significant *Krt14* expression in meibocytes (Fig. S5F). Given the strong transcriptional correlation between *Dhcr24* and *Krt14* in meibocytes (Fig. 2I), we validated their co-localization in young murine MGs via IF co-staining (Fig. 2J). This finding provided evidence for employing the *Krt14* promoter to drive Cre recombinase expression and construct meibocyte-specific *Dhcr24-*cKO model (*Krt14-Cre^ERT^ Dhcr24^fl/fl^*; Schematic diagram of the conditional deletion shown in Fig. S6). Efficient *Dhcr24* knockout was confirmed through IF, WB, and transcriptomics analyses compared to *Dhcr24^fl/fl^* or wild type (WT) controls (Fig. 2K-N).

### Meibocyte-specific* Dhcr24*-cKO mice exhibited marked MGD phenotypes and exacerbated BAC-induced ocular damage

**Figure 3:**
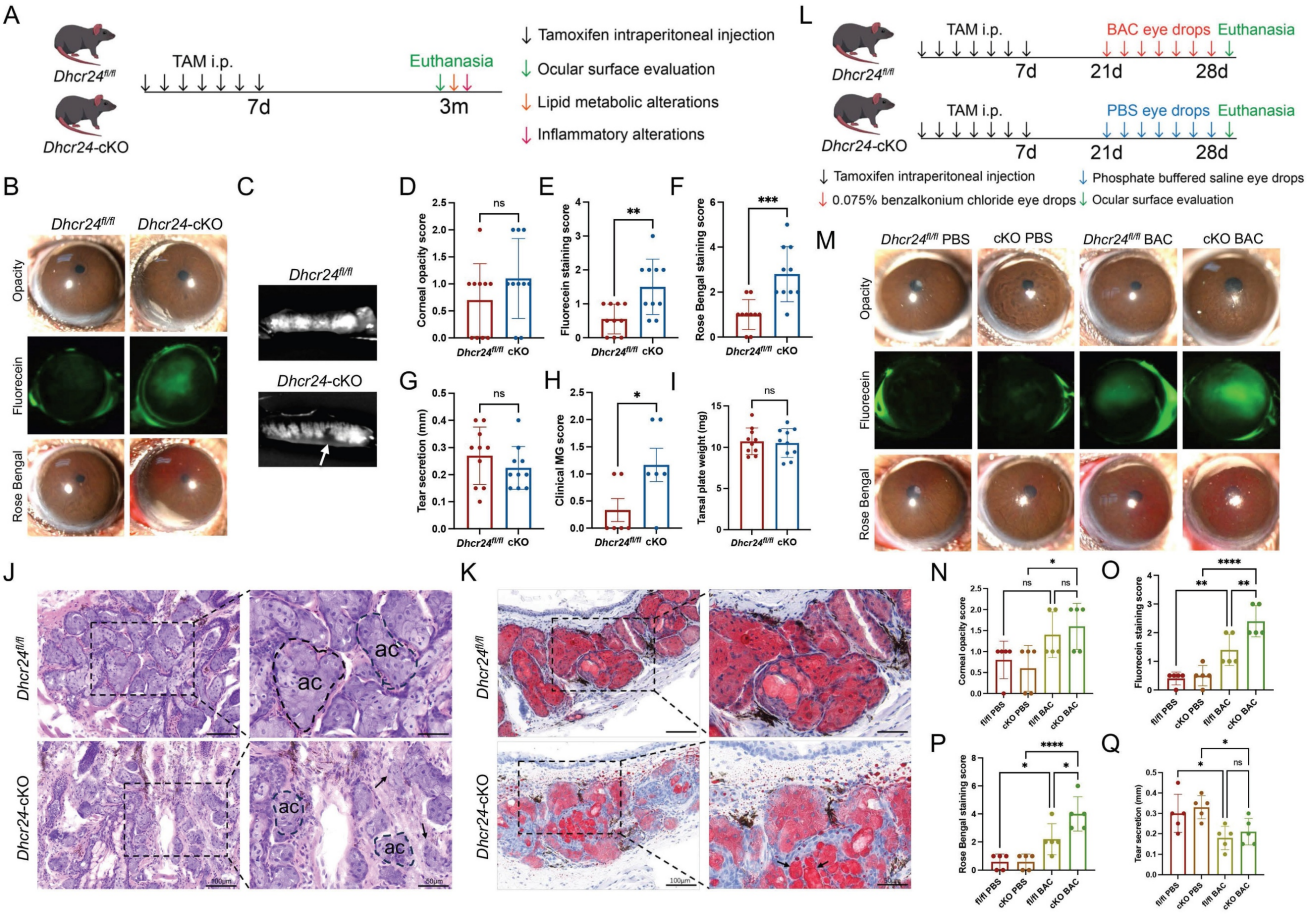
** Phenotypes of meibomian gland dysfunction (MGD) in meibocyte-specific** ***Dhcr24***** conditional knockout (cKO) mice.** (**A**) Experimental timeline for the ocular surface assessments in *Dhcr24*-cKO mice. Two-month-old *Dhcr24*-cKO and *Dhcr24^fl/fl^* mice were injected with tamoxifen (TAM) for 7 days and raised for 3 months before evaluation of ocular surface phenotype and analysis of lipid metabolic and inflammatory alterations. (**B**) Slit lamp images showing representative white light photos, fluorescein staining, and Rose Bengal staining of *Dhcr24^fl/fl^* and *Dhcr24*-cKO mouse corneas. (**C**) Representative images of the upper tarsal plate for *Dhcr24^fl/fl^* and *Dhcr24*-cKO mice, with arrows indicating truncated meibomian glands (MGs). (**D**-**H**) Ocular surface and MG scoring comparisons between *Dhcr24^fl/fl^* and *Dhcr24*-cKO mouse, including (**D**) corneal opacity score (*n* = 10), (**E**) corneal fluorescein staining score (*n* = 10), (**F**) Rose Bengal staining score (*n* = 10), (**G**) relative tear secretion (*n* = 10), and (**H**) clinical MG score (*n* = 6). (**I**) Upper tarsal plate weight comparison between *Dhcr24^fl/fl^* and *Dhcr24*-cKO mice (*n* = 10). (**J**) Hematoxylin-eosin (H&E) staining of the upper tarsal plate, with a dotted circle indicating a single MG acinus (ac) and arrows pointing to disrupted basement membrane and gland structures. (**K**) Oil Red O staining of upper tarsal plate sections, with arrows highlighting aberrant lipid accumulation. (**L**) Experiment timeline for BAC treatment and ocular surface assessments. Two-month-old female *Dhcr24*-cKO and *Dhcr24^fl/fl^* mice were injected with TAM for 7 days and raised for 2 weeks before administered with BAC or phosphate-buffered saline (PBS) eye drops, followed by comprehensive phenotype evaluation. (**M**) Slit lamp images showing representative white light photos, fluorescein staining, and Rose Bengal staining of the *Dhcr24^fl/fl^* with PBS, *Dhcr24*-cKO with PBS, *Dhcr24^fl/fl^* with BAC, and *Dhcr24*-cKO with BAC. (**N**-**Q**) Ocular surface and MG scoring for the treatment groups, including (**N**) corneal opacity score (*n* = 5), (**O**) corneal fluorescein staining score (*n* = 5), (**P**) Rose Bengal staining score (*n* = 5), and (**Q**) relative tear secretion (*n* = 5).

Two-month-old *Dhcr24*-cKO (*n* = 10) and *Dhcr24^fl/fl^* (*n* = 10) mice were treated with TAM and raised for an additional 3 months to allow for phenotype development. Throughout this period, *Dhcr24*-cKO mice exhibited normal growth comparable to their *Dhcr24^fl/fl^* littermates without premature mortality or obvious signs of systemic distress (Fig. S7A). Body weight monitoring revealed no significant differences between the genotypes (Fig. S7B). H&E staining of tissues, including the liver, heart, kidney, spleen, lung, and skin, confirmed normal tissue architecture without inflammation, degeneration, or neoplasia (Fig. S7C), confirming the absence of systemic toxicity or health decline in *Dhcr24*-cKO mice. Comprehensive ocular surface assessments were then conducted using slit-lamp biomicroscopy, corneal fluorescein/Rose Bengal staining, MG morphological analysis, and lipid metabolic/inflammatory profiling (Fig. 3A). Both groups exhibited clear corneas with comparable opacity scores (Fig. 3B, D), but fluorescein staining revealed mild corneal epithelial defects in *Dhcr24*-cKO mice (Fig. 3B), confirmed by significantly elevated fluorescein scores (Fig. 3E). Minimal Rose Bengal staining was observed in *Dhcr24^fl/fl^* mice, while *Dhcr24*-cKO mice showed prominent staining (Fig. 3B, F), reflecting tear film instability and impaired epithelial barrier of the ocular surface. Notably, Schirmer's test demonstrated no differences in basal tear secretion between groups (Fig. 3G), suggesting that epithelial damage in* Dhcr24*-cKO mice arises primarily from abnormal lipid secretion and ocular surface integrity rather than reduced aqueous tear production.

Morphological analysis of upper eyelids revealed clear contrasts in MG structure. *Dhcr24^fl/fl^* mice exhibited well-organized acinar structures, whereas *Dhcr24*-cKO mice demonstrated focal glandular atrophy and ductal dropout (Fig. 3C). These structural abnormalities correlated with significantly elevated MG pathology scores in *Dhcr24*-cKO mice (Fig. 3H), despite comparable total gland weights between groups (Fig. 3I), implying localized rather than systemic gland atrophy. Histological H&E evaluation further confirmed these findings, showing disrupted basement membranes and reduced, irregularly shaped acini in *Dhcr24*-cKO MGs (Fig. 3J). ORO staining highlighted diminished lipid content within *Dhcr24*-cKO acini and aberrant lipid droplet accumulation in ducts (Fig. 3K). Collectively, these results establish that meibocyte-specific *Dhcr24* deletion leads to MGD features, including glandular atrophy, lipid secretion defects, and ocular surface compromise. This phenotype closely resembles ARMGD, emphasizing DHCR24's essential role in preserving MG functional integrity and ocular surface homeostasis.

To further investigate the implications of DHCR24 deficiency, we employed a BAC-induced ocular surface injury model, a well-established approach for studying dry eye disease pathogenesis. Following TAM induction, two-month-old *Dhcr24*-cKO (*n* = 5) and *Dhcr24^fl/fl^* mice (*n* = 5) were administered BAC eye drops twice daily for two weeks, while an equal number of *Dhcr24*-cKO and *Dhcr24^fl/fl^* mice received PBS as the control group (Fig. 3L). After BAC treatment, *Dhcr24*-cKO mice exhibited a relatively opalescent cornea, contrasting with the clear and homogeneous corneas observed in PBS-treated counterparts (Fig. 3M). Quantitative scores confirmed significantly greater corneal opacity in *Dhcr24*-cKO mice following BAC treatment (Fig. 3N), implicating DHCR24 deficiency in worsening chemical stress-induced ocular surface inflammation. Furthermore, fluorescein and Rose Bengal staining revealed severe corneal epithelial defects in BAC-treated *Dhcr24*-cKO mice compared to BAC-treated *Dhcr24^fl/fl^* group (Fig. 3M), with increased scorings reflecting compromised barrier integrity (Fig. 3O, P). Schirmer's test demonstrated comparable basal tear secretion between *Dhcr24^fl/fl^* and *Dhcr24*-cKO mice post-BAC treatment (Fig. 3Q), supporting previous evidence that *Dhcr24* knockout primarily disrupts lipid secretion and ocular surface balance rather than tear production. Notably, the ocular surface pathology in *Dhcr24*-cKO mice was substantially less severe at the early 2-week time point post-TAM induction (Fig. 3N-Q) than after an extended 3-month period (Fig. 3D-G), suggesting that the pathological process requires longer duration to fully develop. Overall, these findings highlight DHCR24's essential role in mitigating chemical stress-induced ocular damage, further linking its deficiency to pathological lipid imbalance and barrier dysfunction.

### DHCR24 deficiency disrupted lipid metabolism and cholesterol homeostasis

**Figure 4:**
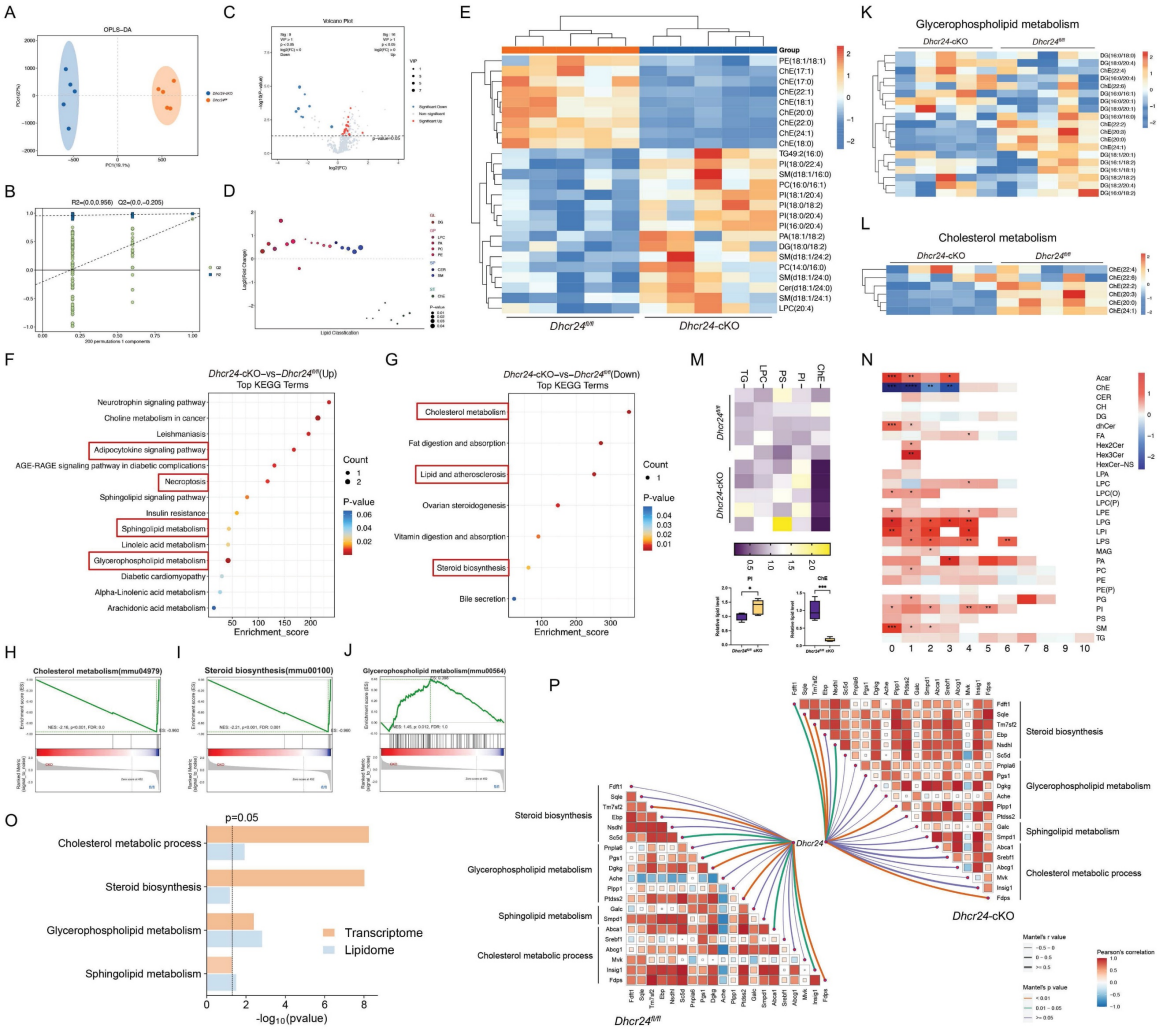
** Comprehensive lipidomics profiling revealing alterations in steroid and cholesterol metabolic process in *Dhcr24* conditional knockout (cKO) mice.** (**A**) Orthogonal partial least squares discriminant analysis (OPLS-DA) and (**B**) OPLS-DA-spot indicating significant trends distinguishing the lipidomics profiling of *Dhcr24^fl/fl^* and *Dhcr24*-cKO groups. (**C**) Volcano plot showing 582 lipid metabolites, in which 16 were significantly upregulated and 9 were downregulated in the *Dhcr24*-cKO vs. *Dhcr24^fl/fl^* group (*p* < 0.05 & VIP > 1). (**D**) Sub class identification of the differentially expressed lipid metabolites between the *Dhcr24*-cKO and *Dhcr24^fl/fl^* groups. (**E**) Heatmap of the 25 differentially expressed lipids. (**F**, **G**) Kyoto encyclopedia of genes and genomes (KEGG) pathway analysis identifying significantly (**F**) upregulated and (**G**) downregulated pathways between the *Dhcr24*-cKO and *Dhcr24^fl/fl^* groups. (**H**-**J**) Gene set enrichment analysis (GSEA) based on lipid metabolites identifying significantly enriched pathways between the *Dhcr24*-cKO and *Dhcr24^fl/fl^* groups, including (**H**) cholesterol metabolism, (**I**) steroid biosynthesis, and (**J**) glycerophospholipid metabolism. (**K**, **L**) Heatmap based on lipid metabolites for significantly enriched metabolic pathways of (**K**) cholesterol metabolism and (**L**) glycerophospholipid metabolism. (**M**, **N**) Comparison of the (**M**) ratio of saturation for ChE, TG, PS, PI, and LPC, and (**N**) total unsaturation patterns between the *Dhcr24*-cKO and *Dhcr24^fl/fl^* groups. ChE, cholesteryl esters; LPC, lysophosphatidylcholine; PI, phosphatidylinositol; PS, phosphatidylserine; TG, triglyceride. (**O**) Significantly enriched signaling pathways identified through integrated transcriptomics and lipidomics of the *Dhcr24*-cKO vs. *Dhcr24^fl/fl^* mice, including cholesterol metabolic process, steroid biosynthesis, glycerophospholipid metabolism, and sphingolipid metabolism. (**P**) Correlation networks between DHCR24 and four significant lipid metabolic pathways in both *Dhcr24*-cKO and *Dhcr24^fl/fl^* mice.

Given DHCR24's pivotal role in cholesterol biosynthesis, we hypothesized that its deficiency would disrupt lipid metabolism in MGs. Untargeted lipidomic profiling of MGs from *Dhcr24*-cKO (*n* = 5) and *Dhcr24^fl/fl^* mice (*n* = 5) revealed significant metabolic divergence between groups via OPLS-DA (Fig. 4A, B). Differential analysis identified 25 significantly altered lipid metabolites (16 upregulated, 9 downregulated; *p* < 0.05, VIP > 1) spanning nine subclasses: PE, ChE, phosphatidylinositol (PI), ceramides (Cer), lysophosphatidylcholine (LPC), phosphatidic acid (PA), SM, DG, and TG (Fig. 4C, D). Notably, a comparison of significantly altered lipid species revealed overlap between aged and *Dhcr24*-cKO conditions, with common dysregulation observed in ChE, DG, PC, PE, SM, and TG classes (Fig. S8A), indicating that DHCR24 deficiency mirrors age-related lipid remodeling. Among the most differentially expressed lipid species, several ChE species (e.g., ChE(18:1), ChE(22:1), and ChE(24:1)) were significantly downregulated in *Dhcr24*-cKO mice, while PI, SM, PC species (e.g., PI(18:0/20:4), SM(d18:1/24:0), and PC(16:0/16:1)) were markedly elevated (Fig. 4E and Fig. S8B). ChE exhibited a negative correlation with other lipid species, suggesting a potential relationship involving their synthesis and transformation (Fig. S8C). These changes suggest a shift away from cholesterol esterification toward altered phospholipid and sphingolipid metabolism, potentially impacting MG lipid secretion and ocular surface stability.

Pathway enrichment analysis substantiated these findings. KEGG mapping highlighted upregulated pathways such as adipocytokine signaling, necroptosis, and sphingolipid/glycerophospholipid metabolism (Fig. 4F), alongside downregulated cholesterol metabolism and steroid biosynthesis (Fig. 4G). Complementary GSEA of transcriptomic data confirmed downregulation in cholesterol biosynthesis, steroid biosynthesis, and glycerophospholipid metabolism (Fig. 4H-J), with heatmaps further visualizing these dysregulated pathways (Fig. 4K, L). To visually integrate the lipidomic alterations observed in *Dhcr24*-cKO mice, a Sankey diagram illustrates the flow from the dysregulated lipid classes (ChE, DG, PA, LPC, PC, PE, PI, SM, TG, Cer) to significantly enriched metabolic and inflammatory pathways, including cholesterol metabolism, glycerophospholipid metabolism, sphingolipid signaling, adipocytokine signaling, the NF-κB signaling pathway, autophagy, efferocytosis, Fc gamma R-mediated phagocytosis, and cAMP signaling (Fig. S8D). Additionally, analysis of lipid saturation revealed significantly reduced levels of saturated ChE species and increased levels of saturated PI species in *Dhcr24*-cKO mice (Fig. 4M), which paralleled lipid compositional shifts observed in aged mice 9. Furthermore, the unsaturation of total ChE and PI was significantly altered (Fig. 4N). Integrated transcriptomic and lipidomic analysis confirmed enrichment of cholesterol/steroid biosynthesis pathways (Fig. 4O), and correlation networks linked *Dhcr24* levels to dysregulated metabolic pathways, including steroid biosynthesis, glycerophospholipid metabolism, and sphingolipid metabolism (Fig. 4P). Collectively, these findings position DHCR24 as a crucial regulator of lipid homeostasis in MGs. Its deficiency disrupts cholesterol metabolism and drives pathogenic lipid redistribution, providing mechanistic insights into lipid-driven pathology in ARMGD.

### Activation of cytokines and inflammatory pathways in *Dhcr24*-cKO mice

**Figure 5:**
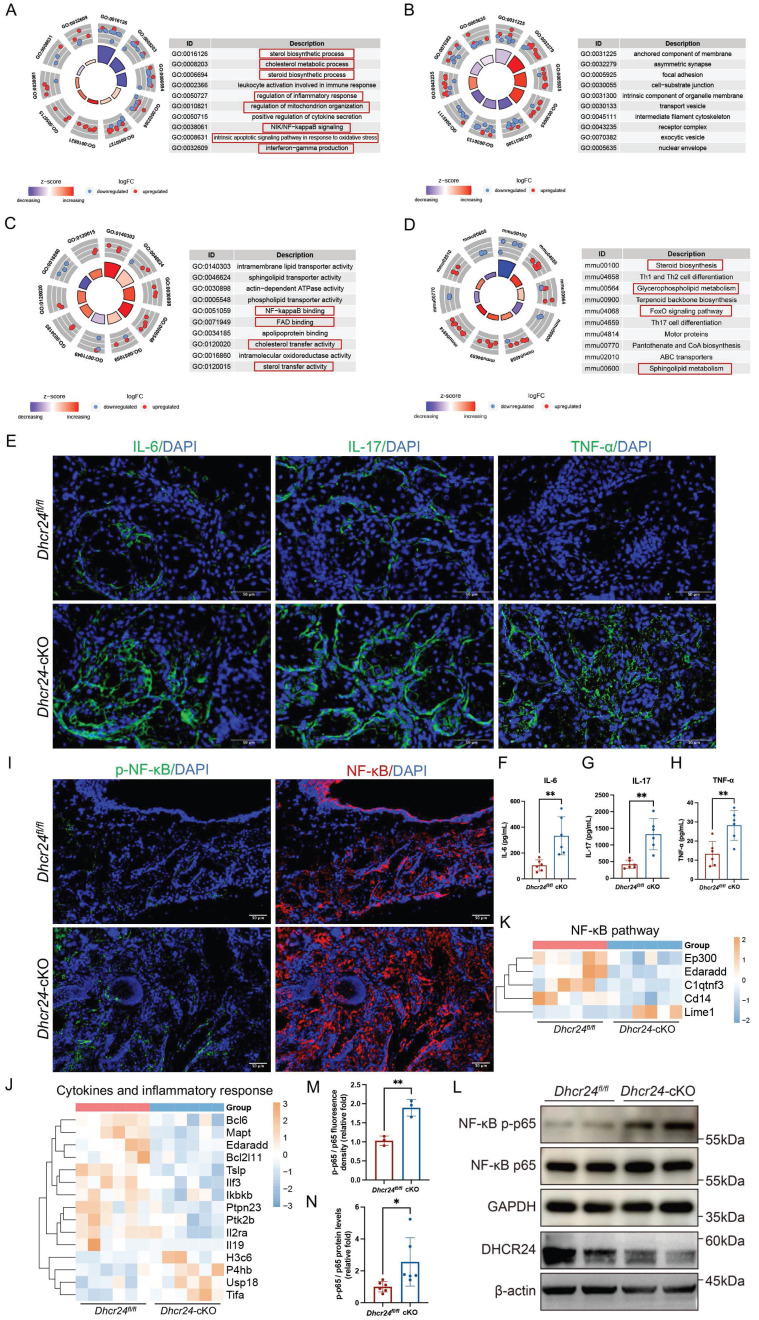
** Activation of cytokines and inflammatory pathways in meibocyte-specific** ***Dhcr24***** conditional knockout (cKO) mice.** (**A**-**C**) Gene ontology (GO) analysis of differentially expressed genes (DEGs) revealing significantly enriched pathways in three categories, including (**A**) biological process (BP), (**B**) cellular component (CC), and (**C**) molecular function (MF). (**D**) Kyoto encyclopedia of genes and genomes (KEGG) enrichment analysis of DEGs between *Dhcr24^fl/fl^* and *Dhcr24*-cKO mice. (**E**) Representative immunofluorescence (IF) images of interleukin-6 (IL-6), interleukin-17 (IL-17), and tumor necrosis factor-α (TNF-α) in *Dhcr24*-cKO vs. *Dhcr24^fl/fl^* mice. Scale bars: 50 μm. (**F-H**) Enzyme-linked immunosorbent assay (ELISA) measurement of meibomian gland IL-6, IL-17, and TNF-α concentrations from *Dhcr24*-cKO and *Dhcr24^fl/fl^* mice. (**I**) Representative IF images of nuclear factor κB (NF-κB) p65 and phospho-NF-κB p65 in *Dhcr24*-cKO vs. *Dhcr24^fl/fl^* group. Scale bars: 50 μm. (**J**, **K**) Heatmap based on differentially expressed genes for significantly enriched pathways of (**J**) cytokines and inflammatory reponse and (**K**) NF-κB signaling. (**L**) Western blot of phospho-NF-κB p65 and NF-κB p65 protein levels. (**M**) Relative fluorescence intensity and (**N**) protein levels of phospho-NF-κB p65 and NF-κB p65.

Transcriptomic profiling of MGs from *Dhcr24*-cKO (*n* = 6) and *Dhcr24^fl/fl^* mice (*n* = 6) three month after TAM treatment revealed significant enrichment in pathways associated with dysregulated lipid metabolism and inflammation. GO analysis highlighted disruptions lipid metabolism, including sterol and steroid biosynthesis and cholesterol metabolic processes, in addition to the activation of the NIK/NF-κB signaling pathway, intrinsic apoptotic signaling in response to oxidative stress, interferon (IFN)-γ production, and regulation of mitochondrial organization (Fig. 5A). Cellular component (CC) and molecular function (MF) analyses further identified enrichment in focal adhesion complexes, organelle membrane components, NF-κB binding activity, and cholesterol/sterol transporter functions (Fig. 5B, C). KEGG confirmed these findings, demonstrating enrichment in steroid biosynthesis, glycerophospholipid metabolism, FoxO signaling, and sphingolipid metabolism (Fig. 5D), highlighting the interplay between lipid dysregulation and inflammatory cascades in MGD pathogenesis.

IF confirmed increased expression of proinflammatory cytokines in *Dhcr24*-cKO MGs, specifically IL-6, IL-17, and TNF-α (Fig. 5E). To quantitatively measure these cytokines, we conducted ELISA on MG tissue lysates, which revealed a significant increase in the protein concentrations of IL-6, IL-17, and TNF-α in *Dhcr24*-cKO mice compared to *Dhcr24^fl/fl^* controls (Fig. 5F-H). Additionally, activation of NF-κB was evidenced by elevated phosphorylation levels of NF-κB p65 and increased p-NF-κB/NF-κB ratios (Fig. 5I, M). Transcriptomic analysis revealed significant alterations in genes related to cytokines and inflammatory response (Fig. 5J), along with notable changes in the expression of NF-κB-associated genes (Lime 1, Cd14, C1atnf3, Edaradd, and Ep300) in *Dhcr24*-cKO mice (Fig. 5K). WB analysis validated these findings, showing markedly increased p-NF-κB protein levels in *Dhcr24*-cKO mice (Fig. 5L, N). Overall, these data suggest that DHCR24 deficiency promotes MGD through chronic inflammation and activation of the NF-κB pathway, linking lipid metabolic dysfunction to immune dysregulation in MG pathology.

### Mitochondrial dysfunction and cGAS-STING activation underlie DHCR24-deficient MG senescence

While mitochondrial morphological and functional alterations have been observed in MGD, the regulatory mechanisms driving these changes remain elusive 36,37. Notably, the release of mtDNA into the cytoplasm has been implicated in triggering inflammation through the cGAS-STING signaling pathway in dry eye disease 33. However, whether mtDNA release and subsequent activation of cGAS-STING similarly contribute to MGD pathogenesis remains unclear and warrants further investigation.

**Figure 6:**
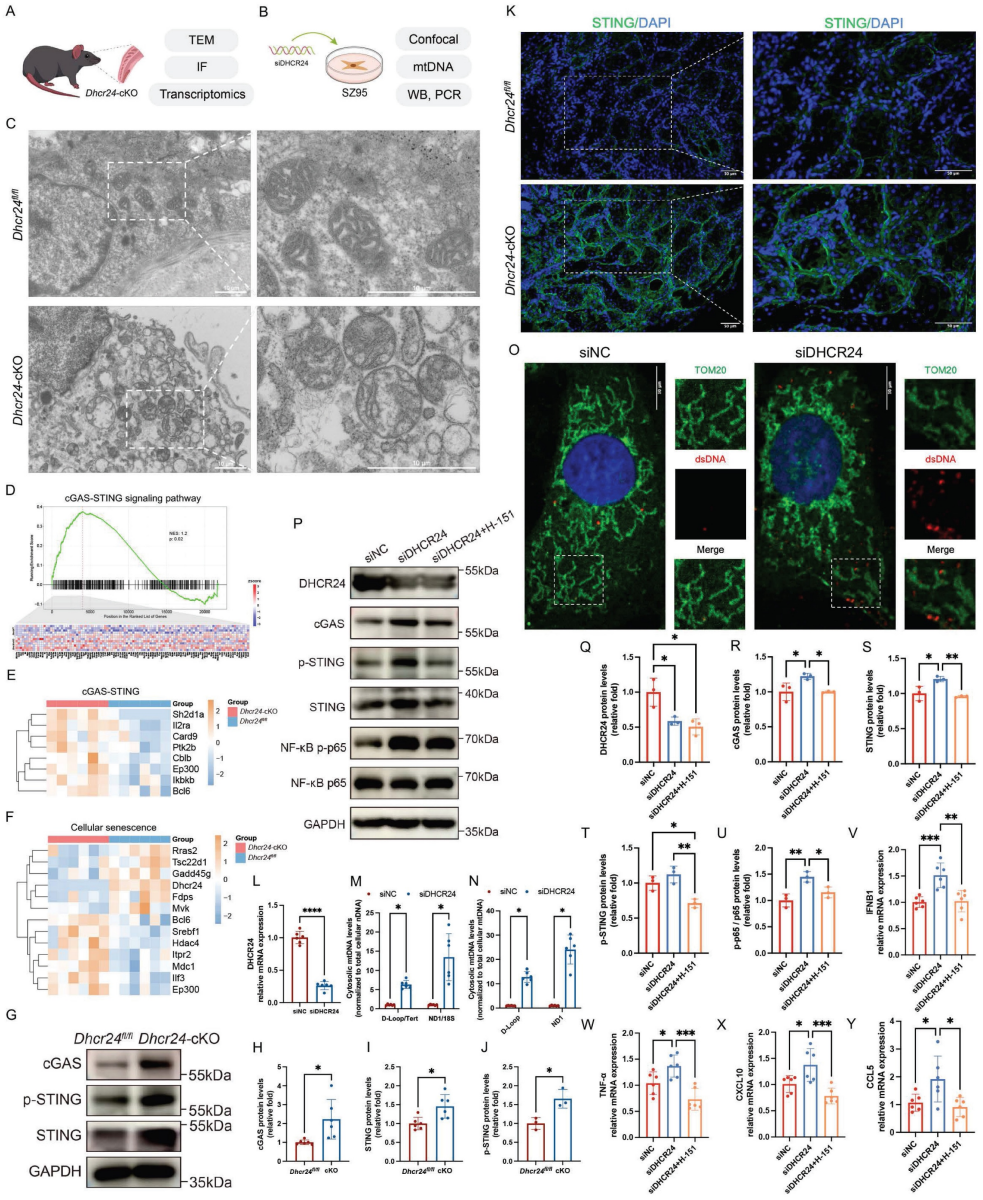
** DHCR24 knockdown induces mitochondrial dysfunction and activates senescence-related cGAS-STING pathway.** (**A**, **B**) Experimental workflow for (**A**) studies in *Dhcr24*-cKO mice and (**B**) *in vitro* experiments in DHCR24-knockdown SZ95 sebocytes. (**C**) Transmission electron microscopy (TEM) revealed mitochondrial swelling and altered cristae structure in meibomian glands (MGs) of *Dhcr24*-cKO mice compared to *Dhcr24^fl/fl^* controls. Scale bars: 10 μm. (**D**, **E**) Gene set enrichment analysis (GSEA) revealed significant enrichment of cGAS-STING signaling pathway in *Dhcr24*-cKO mice, with (**E**) differentially expressed genes (DEGs) shown by a heatmap. (**F**) Heatmap revealed significant enrichment of DEGs associated with cellular senescence. (**G**) Western blot (WB) and relative protein levels of (**H**) cGAS, (**I**) STING, and (**J**) p-STING in *Dhcr24*-cKO vs. *Dhcr24^fl/fl^* MGs. (**K**) Representative immunofluorescence (IF) images of STING in *Dhcr24*-cKO vs. *Dhcr24^fl/fl^* MGs. Scale bars: 50 μm. (**L**) Relative DHCR24 mRNA expression in SZ95 sebocytes treated with control siRNA (siNC) and siDHCR24. (**M, N**) qPCR measurement of (**M**) cytosolic mitochondrial DNA (mtDNA) normalized to total cellular nuclear DNA (nDNA) and (**M**) cytosolic mtDNA normalized to total cellular mtDNA in SZ95 sebocytes treated with siNC and siDHCR24. (**O**) IF for Tom20 and double-stranded DNA (dsDNA) in SZ95 sebocytes treated with siNC and siDHCR24. Scale bars: 10 μm. (**P**) WB for SZ95 sebocytes treated with siNC, siDHCR24, or siDHCR24 plus the STING inhibitor H-151, and relative protein levels were quantified, including (**Q**) DHCR24, (**R**) cGAS, (**S**) STING, (**T**) p-STING, and (**U**) phospho-NF-κB p65 and NF-κB p65. (**V-Y**) Relative mRNA expression of (**V**) IFNB1, (**W**) TNF-α, (**X**) CXCL10, and (**Y**) CCL5 in SZ95 sebocytes treated with siNC, siDHCR24, or siDHCR24 plus H-151.

To address this gap, we investigated mitochondrial dysfunction in DHCR24-deficient MGD models, combining *in vivo* studies in *Dhcr24*-cKO mice (Fig. 6A) with *in vitro* experiments in human sebaceous gland SZ95 cells subjected to DHCR24 siRNA knockdown (Fig. 6B). TEM of *Dhcr24*-cKO MGs revealed swollen mitochondria with fragmented cristae, indicative of severe mitochondrial stress (Fig. 6C). GSEA identified significant dysregulation of the cGAS-STING (Fig. 6D, E) and cellular senescence pathways (Fig. 6F), supported by elevated cGAS, STING, and its activated form p-STING in *Dhcr24*-cKO MGs (Fig. 6G; quantification shown in Fig. 6H-J). IF further demonstrate upregulated STING in *Dhcr24*-cKO MGs (Fig. 6K). Using cytosolic fractionation, we quantified the release of cytosolic mtDNA in DHCR24-knockdown SZ95 sebocytes (Fig. 6L). The cytosolic mtDNA to total cellular nDNA ratio was significantly increased upon DHCR24 knockdown (Fig. 6M). To obtain a direct measurement of mtDNA leakage, we further normalized cytosolic mtDNA to the levels of total cellular mtDNA, which also demonstrated a marked increase (Fig. 6N). Additional IF for Tom20 (a mitochondrial protein) and dsDNA demonstrated that DHCR24 deficiency led to significant accumulation of cytosolic dsDNA (Fig. 6O), providing evidence of mitochondrial membrane destabilization and mtDNA leakage. WB confirmed the activation of both the cGAS-STING and NF-κB pathways upon DHCR24 knockdown, as evidenced by upregulated levels of cGAS, STING, and p-STING in SZ95 sebocytes (Fig. 6P, quantification shown in Fig. 6Q-T). Notably, introduction of the STING inhibitor H-151 in DHCR24-knockdown cells attenuated this activation and reduced downstream NF-κB pathway, indicating that DHCR24 regulates NF-κB signals through the cGAS-STING pathway (Fig. 6P, quantification shown in Fig. 6U). To determine whether cGAS-STING activation led to functional transcription of inflammatory genes, we measured the mRNA levels of key STING-dependent inflammatory and interferon-stimulated genes 38,39. In DHCR24-knockdown SZ95 sebocytes, we observed a significant upregulation of IFNB1, CXCL10, CCL5, and TNF-α (Fig. 6V-Y). Crucially, treatment with H-151 suppressed the induction of all these downstream genes (Fig. 6V-Y). Taken together, these results establish that DHCR24 deficiency induces mitochondrial stress, leading to cytosolic mtDNA release and cGAS-STING-driven inflammation—a mechanism central to MGD-associated senescence and glandular dysfunction.

### AAV-mediated DHCR24 overexpression rescued MG function in aged *Dhcr24*-cKO mice

**Figure 7:**
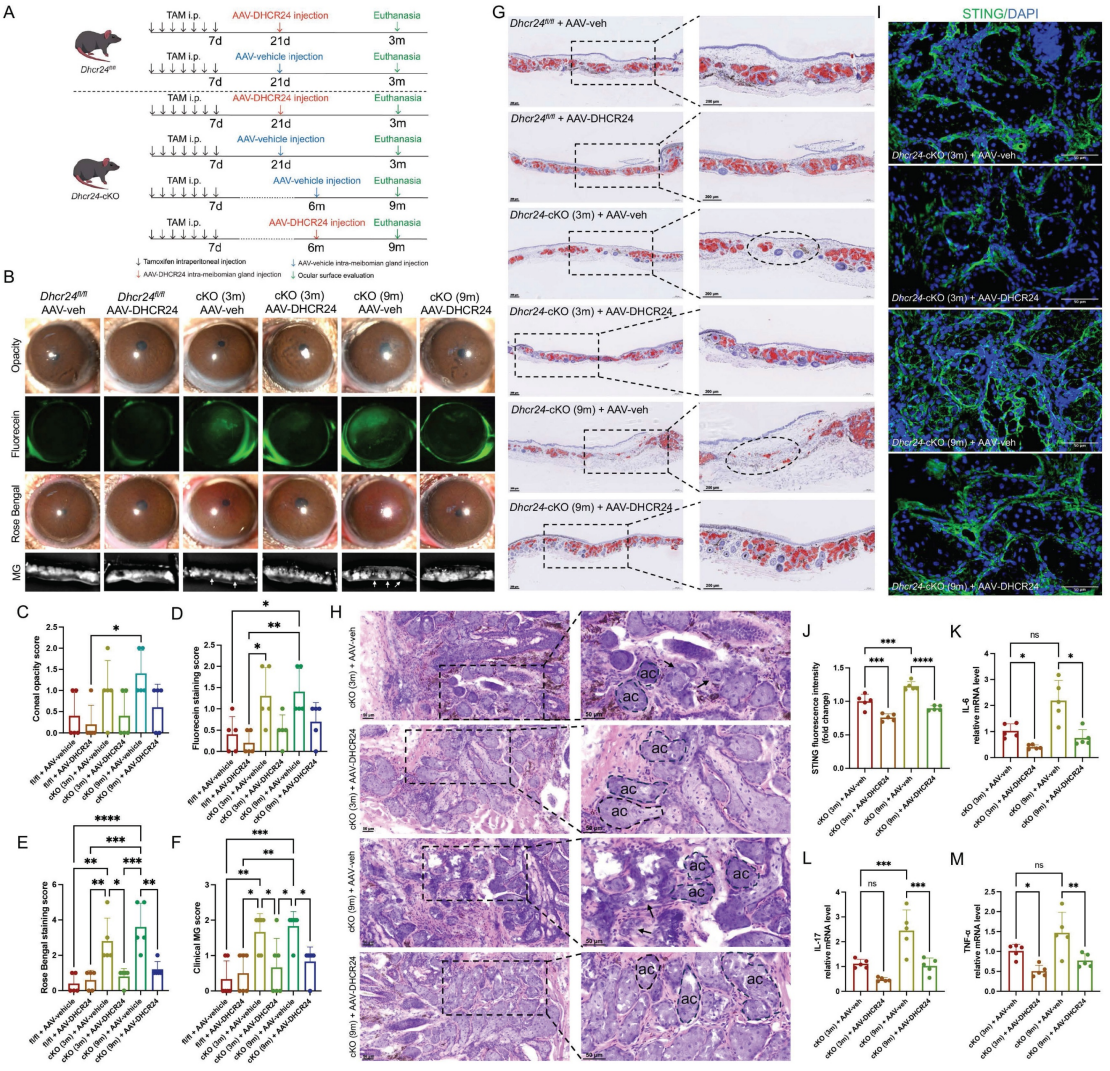
** Adeno-associated virus (AAV)-mediated DHCR24 overexpression reverses ocular surface pathology in *Dhcr24* conditional knockout (cKO) mice.** (**A**) Experiment timeline for AAV treatment and ocular surface assessments. Two-month-old *Dhcr24*-cKO and *Dhcr24^fl/fl^* mice were injected with tamoxifen (TAM) for 7 days and raised for 2 weeks before intra-meibomian gland (MG) injection of AAV-vehicle or AAV-DHCR24, followed by a 3-month period before ocular phenotype evaluation. A separate group of two-month-old *Dhcr24*-cKO mice was raised for 6 months post-TAM before undergoing the same AAV treatment and evaluation procedures. (**B**) Representative white light photos, fluorescein staining, Rose Bengal staining, and upper tarsal plate images for *Dhcr24^fl/fl^*, young *Dhcr24*-cKO (3m), and aged *Dhcr24*-cKO (9m) with AAV-vehicle or AAV-DHCR24 treatment. Arrows pointing to truncated or absent MGs. (**C**-**F**) Ocular surface and MG scoring in the treatment groups, including (**C**) corneal opacity score (*n* = 5), (**D**) corneal fluorescein staining score (*n* = 5), (**E**) Rose Bengal staining score (*n* = 5), and (**F**) clinical MG score (*n* = 5). (**G**) Oil Red O staining of upper tarsal plate sections in the different treatment groups, with circles indicating absent MGs. Scale bars: 200 μm. (**H**) Hematoxylin-eosin (H&E) staining comparing the* Dhcr24*-cKO mice treated with AAV-vehicle and AAV-DHCR24. Scale bars: 50 μm. (**I**) Immunofluorescence (IF) staining and (**J**) relative fluorescence intensity of STING for young *Dhcr24*-cKO (3m) and aged *Dhcr24*-cKO (9m) treated with either AAV-vehicle or AAV-DHCR24. Scale bars: 50 μm. (**K-M**) Relative mRNA expression of (**K**) interleukin-6 (IL-6), (**L**) interleukin-17 (IL-17), and (**M**) tumor necrosis factor-α (TNF-α) in mouse MGs of *Dhcr24*-cKO (3m) and *Dhcr24*-cKO (9m) following AAV-vehicle or AAV-DHCR24 treatment.

To investigate whether restoring *Dhcr24* expression can rescue MG function, we performed intra-MG injections of AAV for *Dhcr24* overexpression in *Dhcr24*-cKO mice. Following TAM induction, two-month-old *Dhcr24*-cKO and *Dhcr24^fl/fl^* mice received either AAV-DHCR24 or AAV-vehicle, followed by a 3-month observation period prior to phenotypic evaluation. Additionally, a separate group of two-month-old *Dhcr24*-cKO mice was raised for 6 months post-TAM before undergoing the same AAV treatment and evaluation procedures (Fig. 7A).

AAV-mediated DHCR24 overexpression in MGs was validated by IF (Fig. S9). Therapeutic intervention via AAV-mediated DHCR24 overexpression demonstrated significant rescue effects in *Dhcr24*-cKO mice. No significant differences in corneal opacity were observed between *Dhcr24*-cKO and *Dhcr24^fl/fl^* mice following AAV injection (Fig. 7B, C). Specifically, intra-MG delivery of AAV-DHCR24 resulted in a substantial reduction of corneal epithelial defects in both young (3m) and aged (9m) *Dhcr24*-cKO mice, as evidenced by diminished fluorescein staining (Fig. 7B, D) and Rose Bengal staining intensities (Fig. 7B, E), suggesting restored corneal epithelial integrity and improved tear film stability. Morphological analyses revealed near-normal MG architecture in AAV-DHCR24-treated *Dhcr24*-cKO mice, characterized by reduced focal dropout and normalized acinar structure (Fig. 7B), confirmed by significantly lowered MG clinical scores (Fig. 7F). Notably, aged (9m) *Dhcr24*-cKO mice exhibited more severe histological features compared to young (3m) counterparts, indicating greater disruption and dropout of acinar morphology (Fig. 7G, H). AAV-mediated DHCR24 overexpression demonstrated significant therapeutic improvements. ORO staining indicated recovery of acinar morphology and lipid droplet content in previously atrophic *Dhcr24*-cKO MGs post-treatment (Fig. 7G). H&E staining further highlighted well-defined basement membranes and a restoration of normal acinar shape in the *Dhcr24*-cKO group after treatment (Fig. 7H).

Importantly, IF analysis revealed a marked reduction in STING expression in both young and aged *Dhcr24*-cKO mice following AAV-DHCR24 treatment (Fig. 7I, J), indicating effective suppression of the STING signaling pathway. Quantitative PCR analysis demonstrated substantial downregulation of pro-inflammatory cytokines, including IL-6, IL-17, and TNF-α, in MGs of AAV-DHCR24-treated mice compared to vehicle-treated controls (Fig. 7K-M). These results suggest that DHCR24 overexpression attenuates MG inflammation and mitigates ocular surface damage by inhibiting the STING pathway and subsequent cytokine production. Overall, our findings highlight the potential of targeted DHCR24 delivery to reverse both structural and functional deficits in aging MGs.

**Figure 8:**
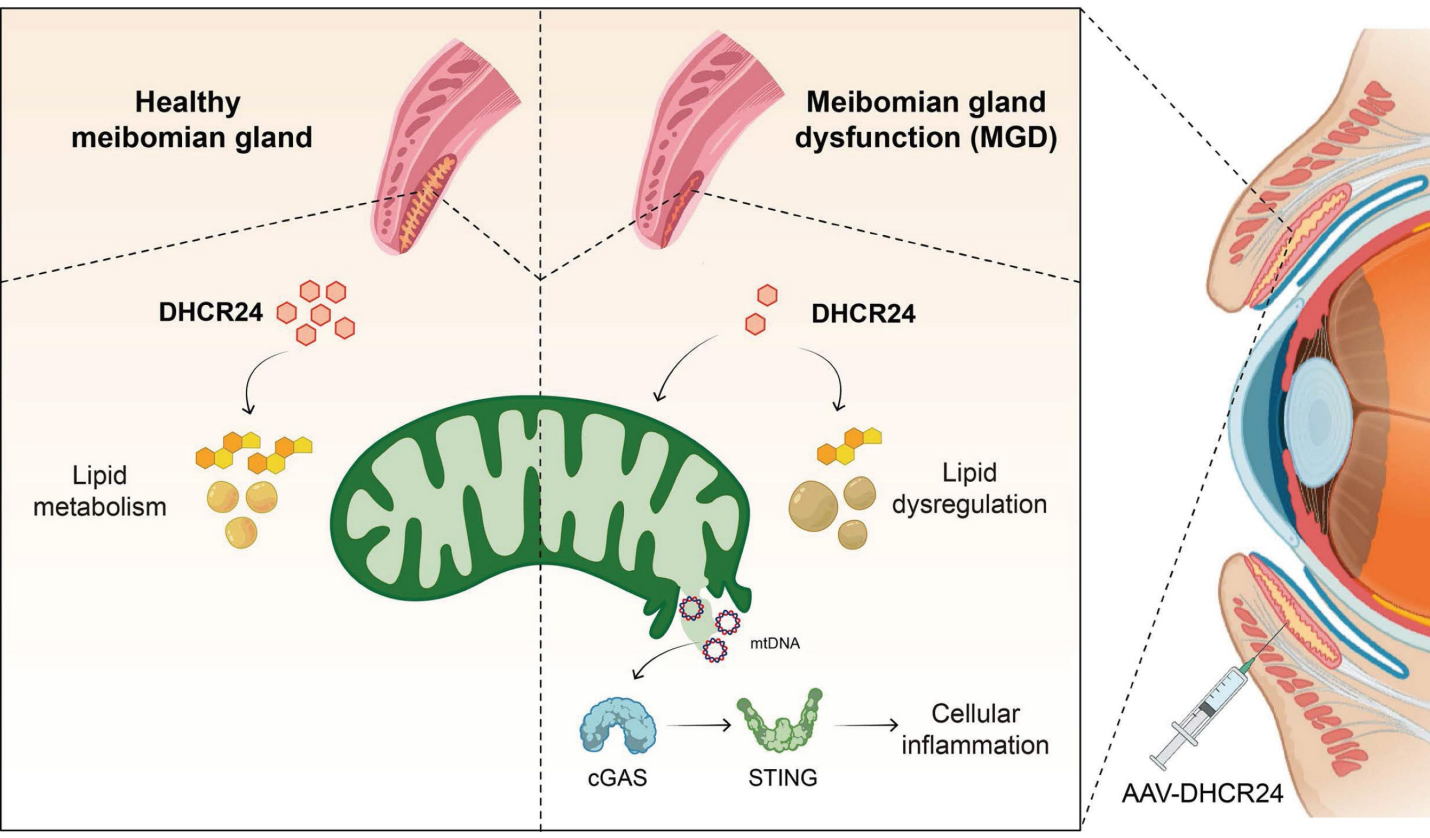
** Graphical mechanism illustrating the role of DHCR24 deficiency in age-related meibomian gland dysfunction (ARMGD).** Deficiency in DHCR24, a key enzyme in cholesterol metabolism, contributes to ARMGD through lipid dysregulation and activation of the cGAS-STING pathway, resulting in chronic cellular inflammation and gland atrophy. Notably, AAV-mediated delivery of DHCR24 restores lipid homeostasis, suppresses STING-dependent inflammatory senescence, and reverses age-related pathological changes. This highlights its potential as a therapeutic strategy for age-related disorders associated with lipid-inflammatory imbalances.

As summarized in the graphical mechanism (Fig. 8), deficiency in DHCR24 contributes to MGD through lipid dysregulation and cGAS-STING-dependent cellular inflammation in MGs. Notably, AAV-mediated delivery of DHCR24 restores lipid homeostasis, suppresses inflammatory senescence, and effectively reverses age-related pathological changes, highlighting its potential as a therapeutic strategy for age-related disorders associated with lipid-inflammatory imbalance.

## Discussion

This study identified the cholesterol metabolism enzyme DHCR24 as a critical regulator of ARMGD through multi-omics screening. Using a meibocyte-specific *Dhcr24* cKO mouse model, we demonstrated that DHCR24 deficiency mimics the pathological features of ARMGD, including ocular surface damage, gland atrophy, lipid metabolic abnormalities, and sustained inflammatory activation. Additional *in vitro* experiments indicated that loss of DHCR24 triggers mitochondrial dysfunction and cytosolic mtDNA leakage, leading to activation of the cGAS-STING signaling pathway, which was further validated in mouse MG tissue. Most significantly, AAV-mediated restoration of DHCR24 reversed age-related MG pathology. These findings establish DHCR24 as a central regulator of lipid metabolism and inflammation in MGs, highlighting its potential as a therapeutic target for ARMGD and other age-related disorders characterized by metabolic-inflammatory imbalance.

Our findings expand the understanding of DHCR24's functional repertoire by revealing its essential role in coordinating lipid metabolism and inflammatory responses during MG aging. DHCR24, a key enzyme in cholesterol biosynthesis, catalyzes desmosterol conversion to cholesterol and regulates cholesterol trafficking and homeostasis 40. Beyond its core lipid metabolic function, its roles span cellular redox regulation, senescence suppression, and inflammatory control across tissues 41. By regulating cholesterol-derived oxidative byproducts, DHCR24 helps maintain redox balance and mitigates oxidative damage, underpinning its therapeutic potential in metabolic disorders such as non-alcoholic fatty liver disease, obesity, and diabetes 42-44, as well as aging-related pathologies like Alzheimer's disease and age-related hearing loss 45. In the brain, age-related DHCR24 decline disrupts cholesterol metabolism, impairing neuronal membrane integrity and synaptic formation 46. Furthermore, its anti-inflammatory and antioxidant properties suppress pro-inflammatory signaling in neurons and microglia, reducing neuroinflammation and oxidative stress 47,48. DHCR24 also intersects with apoptosis pathways, such as Akt/mTOR and Wnt/β-catenin, influencing cell proliferation and stem cell maintenance 49. The pathological changes observed in *Dhcr24*-cKO mice, including disrupted cholesterol metabolism, mitochondrial cristae disorganization, and cytosolic mtDNA leakage, demonstrate that DHCR24 deficiency impairs the metabolic and structural foundations of meibocyte function. The subsequent activation of cGAS-STING signaling and NF-κB-driven inflammation suggests a mechanistic link between cholesterol metabolic disruption and inflammation in ARMGD, representing a previously unrecognized crosstalk in ocular surface pathogenesis. By identifying DHCR24 as a central orchestrator of this lipid-inflammatory axis, our work not only addresses a significant knowledge gap in ocular surface biology but also redefines ARMGD as a disorder of integrated metabolic-inflammatory dysfunction rather than merely an obstructive gland disease. These findings provide a new perspective for understanding and MG aging, positioning DHCR24-mediated pathways as strategic frontiers for therapeutic development in ocular diseases.

The age-related decrease in *Dhcr24* expression reflects a dual mechanism. Consistent with established literature 7,50, our single-cell analysis confirms an age-related decrease in the proportion of meibocytes, implicating mechanisms such as diminished basal cell proliferation and progenitor pool exhaustion in overall cellular loss. Notably, our analysis moves beyond cellular proportion to assess expression at the cluster level. Even after accounting for the reduced number of meibocytes, the cluster-level expression of *Dhcr24* is significantly downregulated in aged MGs compared to young ones. This statistical comparison is based on the mean expression values from all cells within the meibocyte cluster for each group, and its validity is supported by a sufficient cell count in both age groups. Thus, the age-related decline in DHCR24 is not merely a passive consequence of having fewer meibocytes but involves active transcriptional downregulation within the surviving meibocyte population. This downregulation appears to initiate a pathogenic cascade, as evidenced by the close recapitulation of age-associated lipid remodeling in *Dhcr24*-cKO mice. These animals showed a marked reduction in ChE together with alterations in phospholipids, TG, and SM profiles that mirror those observed in aged mice. Furthermore, the model reproduced characteristic shifts in lipid saturation states, including decreased saturated ChE and increased saturated PI species. This finding aligns with published studies demonstrating that the saturation state of meibum lipids shifts with age, particularly in ChE, TG, and PI subclasses 9,51, which are known to impact meibum viscosity and ocular surface stability. In line with clinical observations that ChE unsaturation levels are reduced in MGD and correlate with disease severity 52, our lipidomic analysis revealed significant alterations in total ChE unsaturation patterns between *Dhcr24*-cKO and *Dhcr24^fl/fl^* mice, further supporting the notion that the *Dhcr24*-cKO model captures a key physiochemical alteration relevant to ARMGD. At a systems level, both aging and DHCR24 deficiency converge on common dysregulated pathways, including suppression of cholesterol and steroid biosynthesis, as well as activation of glycerophospholipid metabolism and NF-κB signaling. Collectively, these data identify DHCR24 deficiency as a central driver of the lipid metabolic dysfunction underlying ARMGD.

The cGAS-STING pathway has emerged as a crucial mediator of chronic inflammation in aging tissues 53, yet its specific connection with lipid metabolic dysregulation in ARMGD remains poorly defined. Aging contributes to MG decline through interconnected mechanisms, including lipid dysregulation, oxidative stress, and cellular senescence. Reduced PPAR-γ activity in meibocytes impairs lipid synthesis and secretion, leading to changes in meibum composition, such as shorter fatty acid chains and increased oxidized lipids, which contribute to acinar atrophy 54. Oxidative stress, driven by aging or environmental factors, results in excessive reactive oxygen species (ROS) accumulation, leading to lipid peroxidation, DNA damage, and accelerated MG aging 55,56. Senescent meibocytes release senescence-associated secretory phenotype (SASP) factors, disrupting the balance of proliferation, differentiation, and secretion, and causing structural abnormalities in acinar and ductal systems 57,58. A key factor in these processes is mitochondrial dysfunction, particularly critical in energy-intensive meibomian epithelial cells, though its regulatory mechanisms have remained elusive 59,60. In dry eye disease, corneal epithelial mitochondrial dysfunction correlates with impaired antioxidant defenses and NF-κB-driven inflammation 61,62. Additionally, ROS-induced mitochondrial permeability transition pore (mPTP) may also trigger cGAS-STING pathway activation via mtDNA leakage, amplifying SASP-driven senescence 33. Our data establish that DHCR24 deficiency in ARMGD models leads to concurrent dysregulation of cholesterol metabolism, mitochondrial impairment, and cGAS-STING-driven inflammation. We observed mitochondrial damage with cristae disorganization in *Dhcr24*-cKO mice, while* in vitro* DHCR24 knockdown promoted cytosolic mtDNA leakage and activated cGAS-STING signaling. Further STING inhibition with H-151 in DHCR24-knockdowned SZ95 cells suppressed both cGAS-STING pathway activity and downstream NF-κB-mediated inflammation. Notably, the subsequent reduction in STING levels following AAV-mediated restoration of DHCR24 aligns with the dynamic regulation of this pathway. In the *Dhcr24*-cKO model, constitutive mtDNA leakage likely serves as a persistent ligand driving cGAS-STING activation, which can lead to the transcriptional upregulation and stabilization of STING protein 63. We hypothesize that the restoration of DHCR24 mitigates upstream mitochondrial damage and mtDNA leakage, thereby removing the chronic activating signal. In the absence of this signal, STING may then be subject to normal protein turnover and degradation mechanisms, such as autophagy-lysosomal clearance, allowing its expression to return to baseline 64. While these results position DHCR24 deficiency as a critical node associated with this interconnected pathology, the precise mechanistic link between disrupted cholesterol metabolism and the loss of mitochondrial integrity requires further clarification.

The structural and functional compromise of mitochondria observed in *Dhcr24*-cKO models points to a critical mechanistic link in meibocyte senescence. We propose that DHCR24 deficiency disrupts cholesterol homeostasis, serving as a primary driver of mitochondrial membrane instability. This "lipid-mitochondria-inflammation" axis is supported by recent evidence characterizing mitochondria as pivotal signal relays that translate metabolic dysregulation into cGAS-STING activation 23. We hypothesize that DHCR24 deficiency alters the sterol-to-phospholipid ratio, increasing membrane fragility and facilitating mitochondrial permeabilization or aberrant opening of the mPTP. Notably, inhibition of the cholesterol biosynthetic enzyme ACLY has been shown to induce polyunsaturated fatty acid (PUFA) peroxidation and mitochondrial damage, subsequently triggering mtDNA-mediated cGAS-STING activation 65. Furthermore, enzymes in the cholesterol pathway, such as squalene epoxidase (SQLE), can localize to mitochondria to stabilize mitochondrial transcription factor A (TFAM) and preserve organelle integrity 66. Moreover, the interplay between lipid metabolism and innate immunity involves complex molecular crosstalk that extends beyond mitochondrial stress. Recent studies demonstrate that LXR-mediated lipid metabolism induces enzymes such as SMPDL3A, which acts as a cGAMP-degrading enzyme to restrict cGAS-STING DNA sensing 22,67. This highlights the presence of endogenous feedback mechanisms that may be compromised in aging or DHCR24-deficient states, further promoting a shift toward chronic inflammation.

Beyond elucidating disease mechanisms, our findings provide a translational framework consistent with the urgent clinical need for targeted therapies in ARMGD. Current management strategies are mainly palliative and unable to prevent glandular atrophy or address the underlying metabolic-inflammation imbalance. Our findings establish a foundation for a more targeted therapeutic approach: restoring DHCR24 activity to correct the metabolic disruption and preserve mitochondrial integrity, combined with cGAS-STING inhibitors to attenuate the downstream chronic inflammatory cascade. Preclinical studies support the effectiveness of STING inhibitors in treating ocular surface inflammation 33,68. A combined regimen that simultaneously normalizes lipid metabolism and reduces innate immune activation may exhibit synergistic benefits. This strategy is consistent with the emerging framework of "metabolic-immune" therapies in age-related disorders. However, several challenges must be addressed for clinical translation. Current research on DHCR24 has primarily focused on inhibitors 69,70, while the development of agonists remains underexplored due to the enzyme's complex transmembrane structure 71. Future work should prioritize the development of tissue-specific delivery systems, such as CRISPR activation (CRISPRa) 72, to safely upregulate DHCR24 expression. Additionally, the upstream mechanisms driving age-related DHCR24 downregulation, whether driven by epigenetic modifications, transcriptional silencing, or age-related shifts in the cellular microenvironment, require systematic investigation. Further validation in human MG samples correlating DHCR24 expression with clinical severity will be essential for translational applications. These findings not only enhance our understanding of ARMGD but also create a foundation for addressing metabolic-inflammatory interactions in other age-related conditions, opening new avenues for therapeutic intervention within the broader scope of aging research.

It is noteworthy that this study utilized female mice. Although human epidemiological studies suggest a higher prevalence of dry eye disease in women, of which MGD is a major component, sex-specific prevalence for MGD remains inconsistently reported and is confounded by factors such as age and diagnostic criteria 73,74. In C57BL/6J mice, young adults show no significant sex-based differences in baseline meibomian lipid profiles or gene expression, supporting the use of either sex for fundamental MG research 75. We selected female mice for several reasons: (1) to reduce biological variation introduced by sex as a variable, thereby enhancing the sensitivity of our integrated multi-omics screen; (2) to avoid ocular surface trauma resulting from male-male aggression, which could confound MGD phenotyping; and (3) to prevent potential complications from androgen fluctuations that may influence lipid secretion and gland homeostasis.

This study has several limitations. First, the human tissue analysis was restricted to two samples from non-MGD donors (a 3-year-old and a 40-year-old), due to the significant practical challenge of obtaining human tarsal plate specimens. While this preliminary validation shows an age-associated decline in DHCR24 expression that aligns with our murine findings, the small sample size and lack of tissues from clinically diagnosed ARMGD patients prevent a definitive correlation between DHCR24 loss and human disease pathology. Future studies that include a larger cohort of well-characterized ARMGD patient samples will be essential to establish the clinical relevance and translational potential of DHCR24 as a therapeutic target. Second, while we hypothesize that disrupted cholesterol homeostasis serves as an upstream trigger for mitochondrial stress, the intricate link between these processes remains to be fully elucidated. Future investigations utilizing targeted lipidomics, Seahorse bioenergetic assays, and super-resolution imaging are necessary to define the specific ChE species or cholesterol metabolites and biophysical mechanisms responsible for compromising mitochondrial integrity in ARMGD. These candidate metabolites should then be functionally validated through rescue experiments, such as supplementation in DHCR24-deficient SZ95 sebocytes, to determine whether they can prevent or reverse mitochondrial damage and mtDNA leakage. Defining the functional consequences of these lipid mediators will be crucial in establishing a causal pathway from cholesterol dysregulation to mitochondrial dysfunction. Third, while our data support the hypothesis that AAV-DHCR24 restoration alleviates inflammation by mitigating mitochondrial damage and mtDNA-driven cGAS-STING activation, experimental confirmation that this intervention reduces cytosolic mtDNA leakage *in vivo* is still lacking. Future studies employing* in vivo* imaging or direct quantification of cytosolic mtDNA in MG tissue before and after AAV-DHCR24 treatment would provide crucial mechanistic validation of this proposed sequence of events.

## Conclusion

In summary, this study identifies the cholesterol metabolism enzyme DHCR24 as a critical regulator of ARMGD through multi-omics screening. Utilizing meibocyte-specific *Dhcr24*-cKO mice, we observed MGD phenotypes, including glandular atrophy and aberrant lipid secretion. Further experiments demonstrated that DHCR24 deficiency disrupts lipid metabolism and cholesterol homeostasis and leads to the activation of cytokines and inflammatory pathways in *Dhcr24*-cKO mice. *In vitro* studies with DHCR24-knockdown SZ95 sebocytes demonstrated mtDNA leakage and subsequent activation of the cGAS-STING pathway. Remarkably, AAV-mediated restoration of DHCR24 effectively reversed age-related ocular surface pathology and restored MG integrity. Collectively, these findings establish DHCR24 as a central therapeutic target for lipid dysregulation to inflammation in MGD, highlighting its potential for treating related age-related conditions.

## Supplementary Material

Supplementary figures and table.

## Data Availability

The datasets used and analyzed during the current study are available from the corresponding authors upon reasonable request.
