# Supplementary material for: DHCR24 Deficiency Drives Age-Related Meibomian Gland Dysfunction by Regulating Lipid Metabolic Imbalance and Cytosolic mtDNA-Induced cGAS-STING Activation
Source: Int J Biol Sci. 2026 Mar 28;22(7):3886–908. doi: 10.7150/ijbs.129636 (PMC13086210; doi:10.7150/ijbs.129636)
Supplement: Supplementary file 1 — Supplementary figures and table. [file ijbsv22p3886s1.pdf]

Supplementary Materials for

DHCR24 Deficiency Drives Age-Related Meibomian Gland Dysfunction by Regulating Lipid Metabolic Imbalance and Cytosolic mtDNA-Induced cGAS-STING Activation

Yuchen Cai *et al.*

*Corresponding author: Hao Sun, sunhao6666@126.com (HS); Yao Fu, fuyao@sjtu.edu.cn; Liangbo Chen, chenliangbo@sjtu.edu.cn

This PDF file includes:

Supplementary Figures S1 to S9
Supplementary Table S1

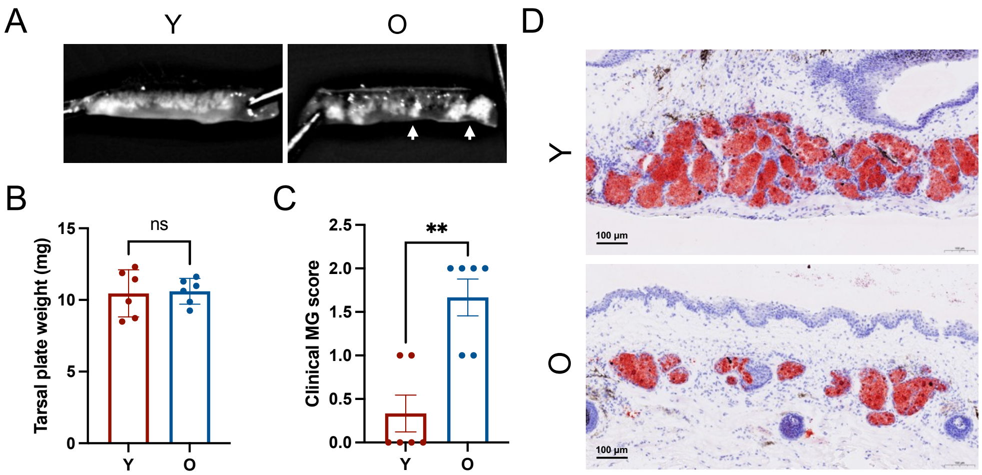

Fig. S1. Assessment of meibomian glands (MGs) in mice between the young (Y) and aged (O) group. (**A**) Representative upper tarsal plate images for Y and O mouse. Arrow indicated MG dropout and disordered MGs. (**B**) Tarsal plate weight. (**C**) Clinical MG scores. (**D**) Oil Red O (ORO) staining of frozen upper eyelid sections. Scale bars, 100 μm.

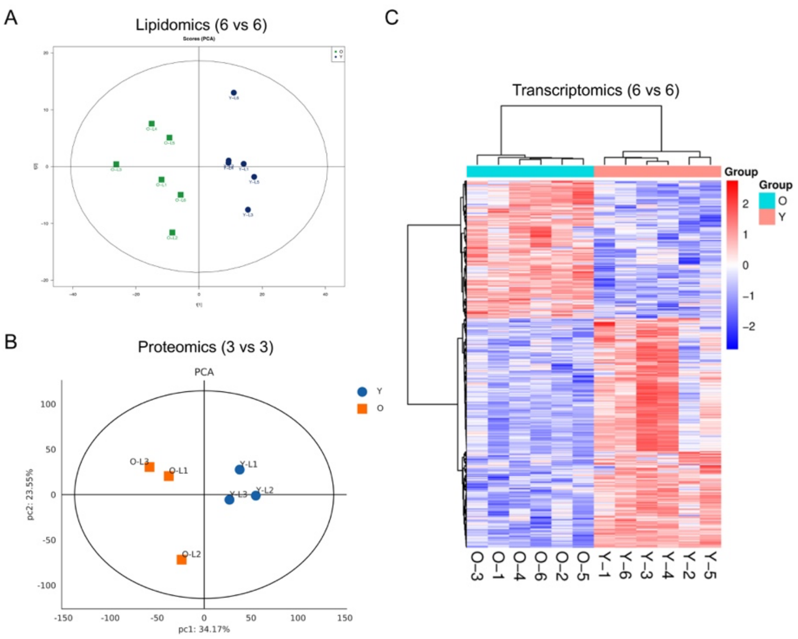

Fig. S2. (**A**, **B**) Principal component analysis (PCA) of (**A**) lipidomics and (**B**) proteomics revealing a significant trend distinction between the young (Y) and aged (O) group. (**C**) Heatmap of upregulated and downregulated differentially expressed genes (DEGs) between the two age groups.

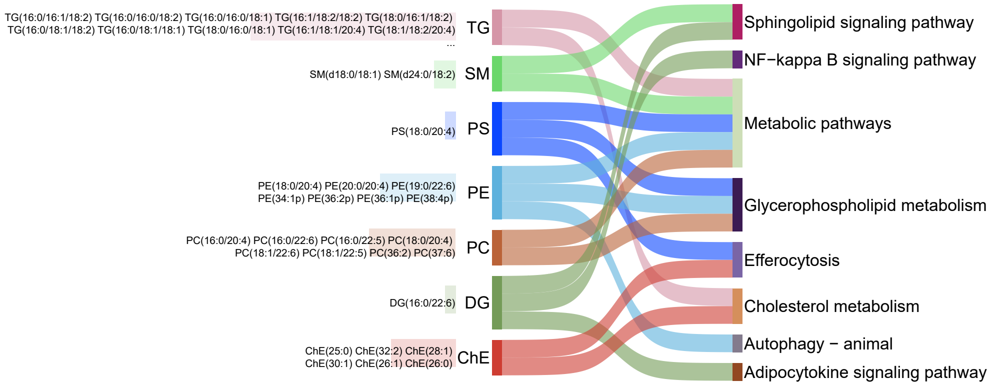

Fig. S3. Sankey diagram illustrating the connection between dysregulated lipid metabolism and inflammatory signaling in aged meibomian glands (MGs). The left layer represents lipid classes and key lipid species that are significantly expressed in aged vs. young mice, as identified by lipidomics. The right layer depicts KEGG pathways that are significantly enriched by the differentially expressed lipids.

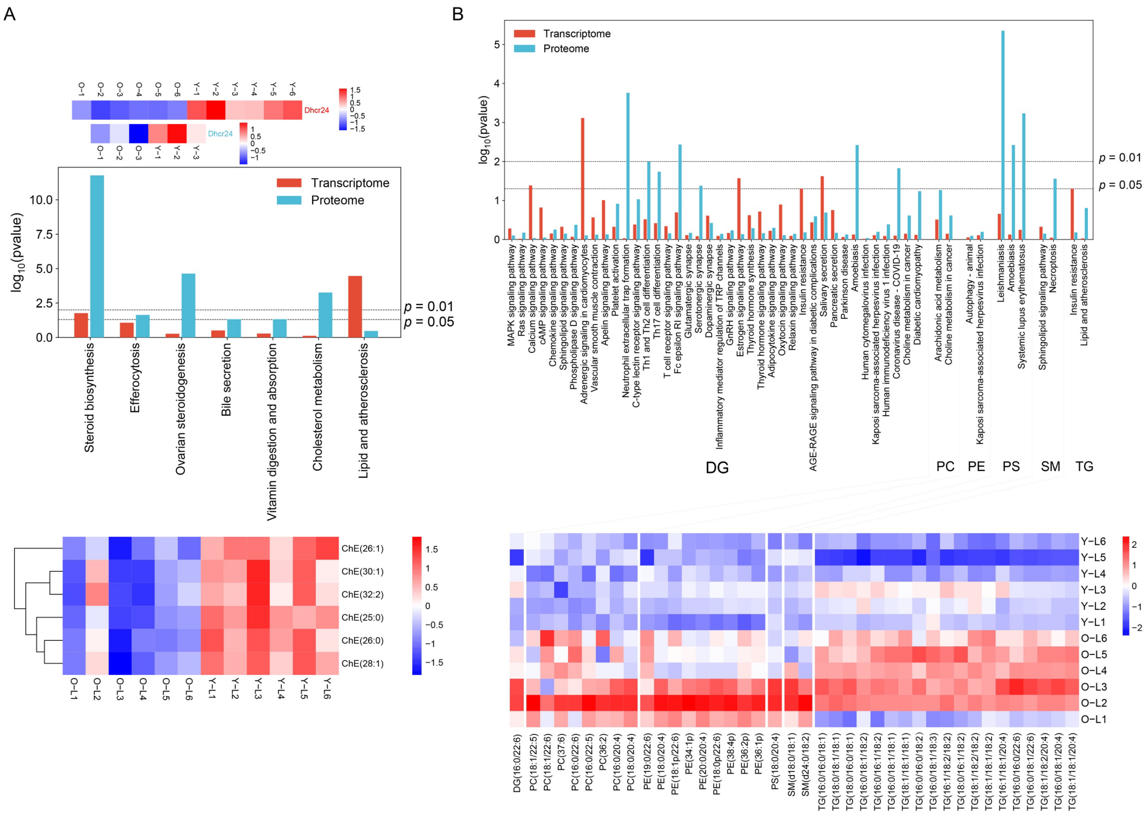

Fig. S4. Integrated multi-omics analysis reveals dysregulated lipid-inflammatory network in ARMGD. (**A**) Analysis of downregulated lipid classes (cholesteryl esters, ChE). Significantly enriched KEGG pathways linked to these lipids are displayed, along with corresponding pathway enrichment observed in the upper section for transcriptomic and proteomic data. (**B**) Analysis of upregulated lipid classes, including diacylglycerols (DG), phosphatidylcholines (PC), phosphatidylethanolamines (PE), phosphatidylserines (PS), sphingomyelins (SM), and triacylglycerols (TG). Their enriched metabolic and inflammatory pathways are presented, with supporting evidence from transcriptomic and proteomic analyses illustrated in the upper section.

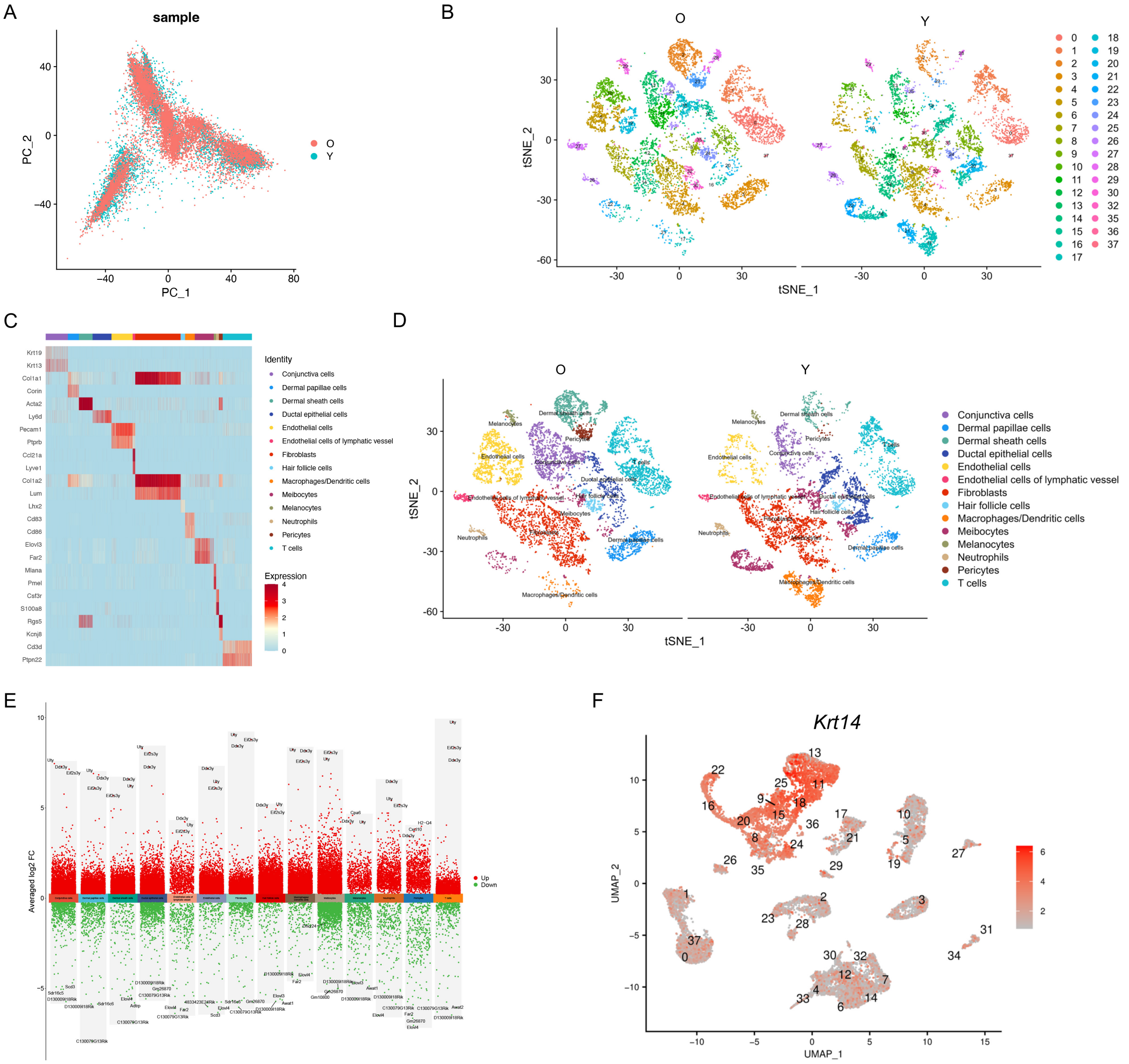

Fig. S5. (**A**) Principal component analysis (PCA) for young (Y) and aged (O) mice, illustrating the linear dimensional reduction of single cell sequencing data. (**B**) t-distributed Stochastic Neighbor Embedding (t-SNE) analysis classifying meibomian gland cells into 37 distinct clusters. (**C**) Annotation of cell groups based on the marker genes of each cell subpopulation. (**D**) t-SNE annotation identifying 14 major cell types within the meibomian gland. (**E**) Differentially expressed genes (DEGs) between the two age groups analyzed for each cell type. (**F**) Scatter plot of *Krt14* expression in each cell cluster analyzed by uniform manifold approximation and projection (UMAP).

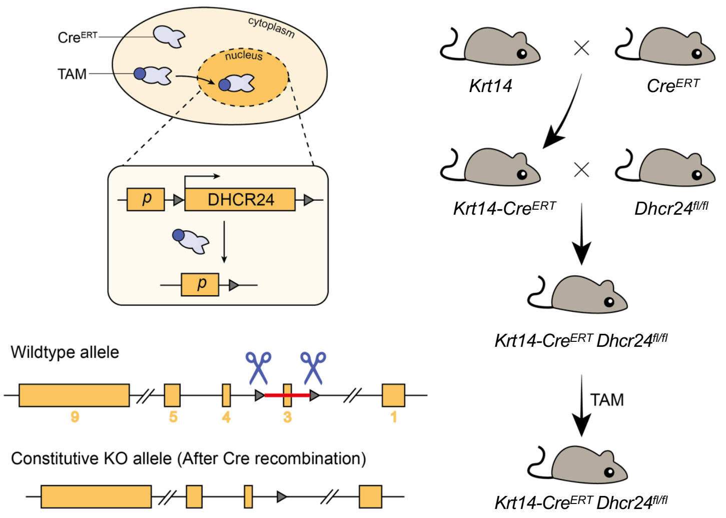

Fig. S6. Schematic diagram of tamoxifen (TAM)-induced conditional deletion of the *Dhcr24* gene in mouse meibomian glands (MGs) via the Cre-LoxP system. In meibocytes, the expression of Cre^{ERT} is driven by the *Krt14* promote. After TAM treatment, Cre^{ERT} binds to TAM and translocates to the nucleus, leading to the specific knockout of the *Dhcr24* gene (exon 3). *Dhcr24$^{fl/fl}$* mice with *Krt14*-Cre^{ERT} mice were crossed to construct meibocyte-specific knockout mice (*Krt14-Cre^{ERT} Dhcr24$^{fl/fl}$*, *Dhcr24*-cKO) for *Dhcr24,* and littermate Cre-negative mice (*Dhcr24$^{fl/fl}$*) served as controls.

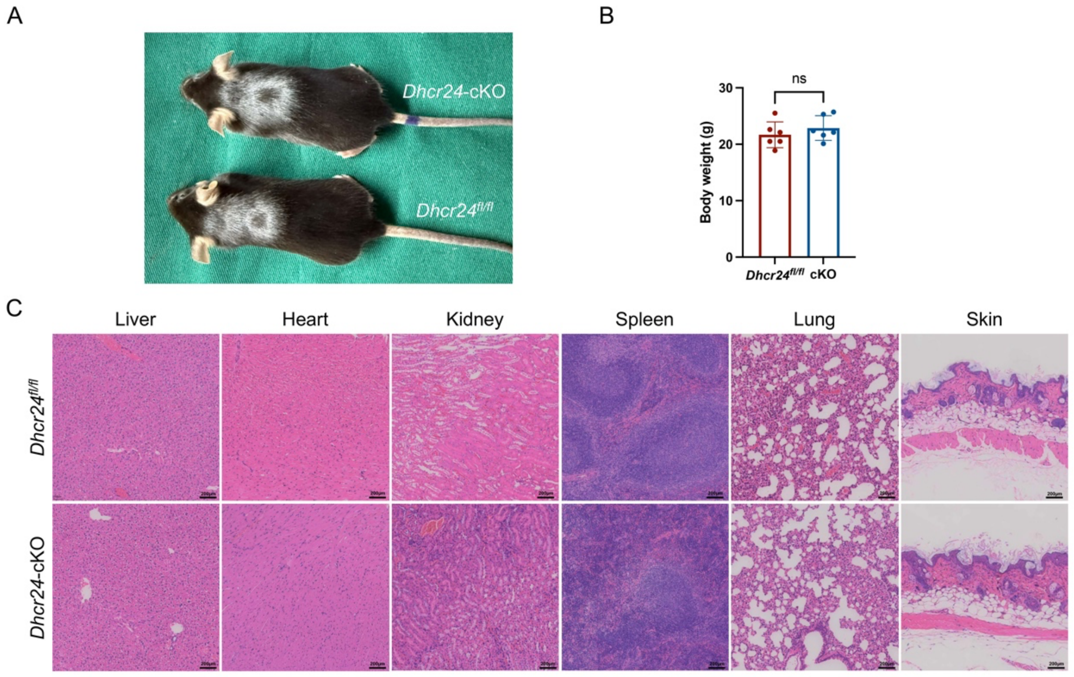

Fig. S7. *Dhcr24*-cKO mice exhibit normal systemic health. (A) Representative photograph showing the gross appearance of *Dhcr24*-cKO and *Dhcr24$^{fl/fl}$* mouse three months post-tamoxifen induction. (B) Body weight of *Dhcr24*-cKO and *Dhcr24$^{fl/fl}$* mouse three months post-tamoxifen induction. Data are presented as mean ± SD. (C) Representative H&E staining of the liver, heart, kidney, spleen, lung, and skin from *Dhcr24*-cKO and *Dhcr24$^{fl/fl}$* mice three months post-tamoxifen induction. Scale bars, 200 μm.

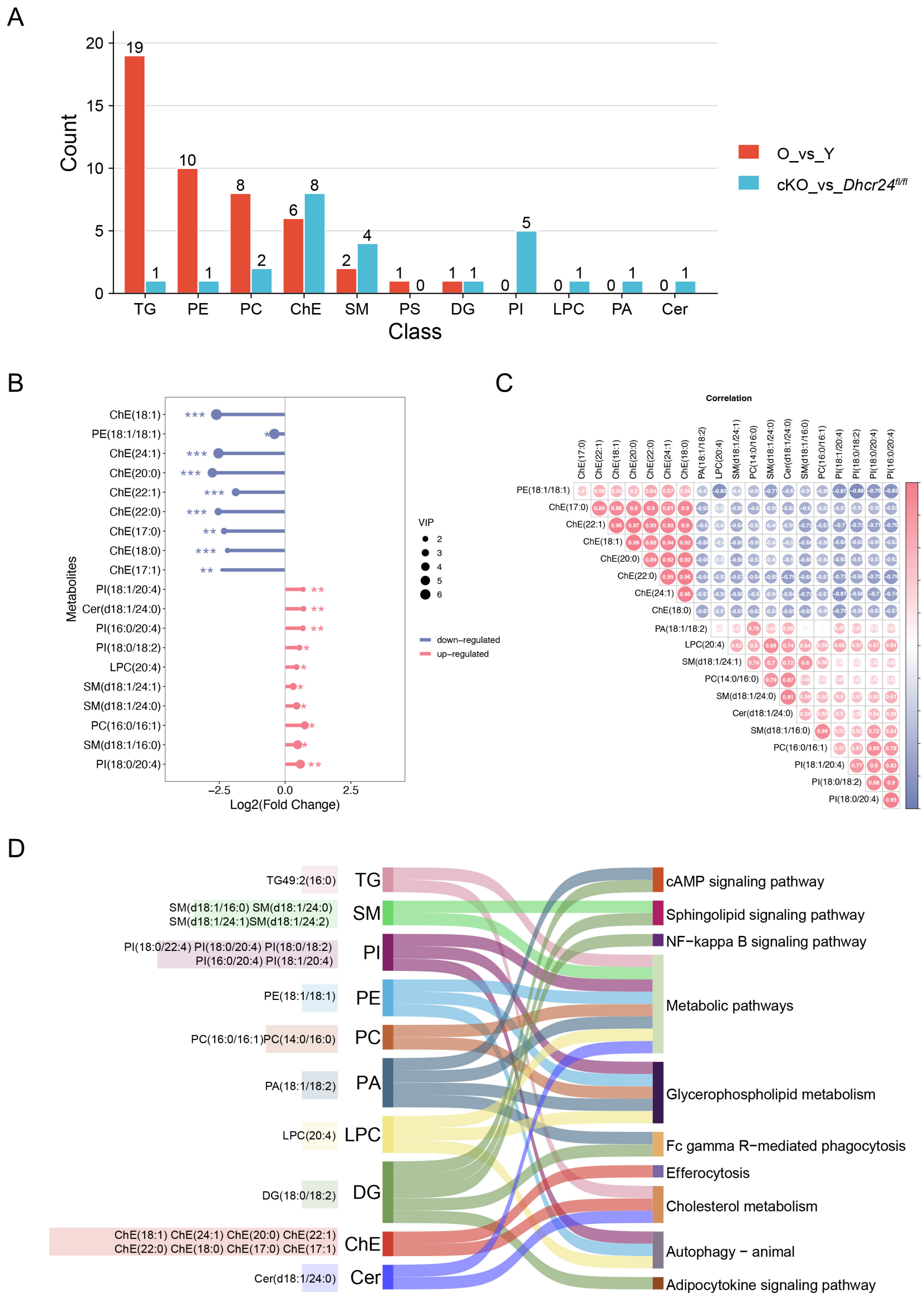

Fig. S8. (**A**) Bar plot comparing the number of significantly expressed lipid species within major lipid classes between two conditions: age-related change (aged vs. young) and *Dhcr24* deficiency (*Dhcr24*-cKO vs. *Dhcr24*$^{fl/fl}$). (**B**) Top 10 significantly upregulated and downregulated lipid metabolites between the *Dhcr24*-cKO vs. *Dhcr24*$^{fl/fl}$ group. (**C**) Correlational analysis for the 25 differentially expressed lipids. (**D**) Sankey diagram illustrating the connection between dysregulated lipid metabolism and inflammatory signaling in *Dhcr24*-cKO vs. *Dhcr24*$^{fl/fl}$ mice. The left layer represents lipid classes and key lipid species that are significantly altered in *Dhcr24*-cKO vs. *Dhcr24*$^{fl/fl}$ mice, as identified by lipidomics. The right layer depicts KEGG pathways that are significantly enriched by the differentially expressed lipids.

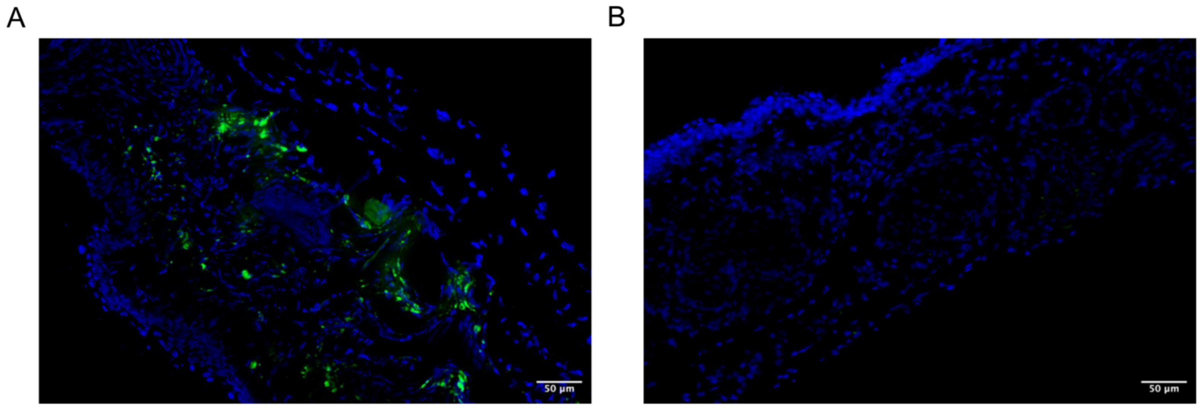

Fig. S9. The tarsal plate cryosection after intra-meibomian gland injections of (**A**) AAV-DHCR24 or (**B**) saline solution for 2 weeks in *Dhcr24*-cKO mice. The nuclei were counterstained with DAPI (blue). Scale bars, 50 μm.

Table S1. Primers used in this study

Primer	Sequences (5'-3')
mouse-IL-6 (F)	TACCACTTCACAAGTCGGAGGC
mouse-IL-6 (R)	CTGCAAGTGCATCATCGTTGTTC
mouse-IL-17 (F)	CAGACTACCTCAACCGTTCCAC
mouse-IL-17 (R)	TCCAGCTTTCCCTCCGCATTGA
mouse-TNF-α (F)	GGTGCCTATGTCTCAGCCTCTT
mouse-TNF-α (R)	GCCATAGAACTGATGAGAGGGAG
mouse-GAPDH (F)	GTGGAGTCATACTGGAACATGTAG
mouse-GAPDH (R)	AATGGTGAAGGTCGGTGTG
human-DHCR24 (F)	CTCTGGGTGCGAGTGAAGG
human-DHCR24 (R)	TTCCCGGACCTGTTTCTGGAT
human-IFNB1(F)	ATGACCAACAAGTGTCTCCTCC
human-IFNB1(R)	GGAATCCAAGCAAGTTGTAGCTC
human-CXCL10(F)	GTGGCATTCAAGGAGTACCTC
human-CXCL10(R)	TGATGGCCTTCGATTCTGGATT
human-CCL5(F)	CCAGCAGTCGTCTTTGTCAC
human-CCL5(R)	CTCTGGGTTGGCACACACTT
human-TNF-α(F)	GAGGCCAAGCCCTGGTATG
human-TNF-α(R)	CGGGCCGATTGATCTCAGC
human-GAPDH (F)	ACAACTTTGGTATCGTGGAAGG
human-GAPDH (R)	GCCATCACGCCACAGTTTC
human-mt-ND1 (F)	CACCCAAGAACAGGGTTTGT
human-mt-ND1 (R)	TGGCCATGGGTATGTTGTTAA
human-mt-DLOOP (F)	CTATCACCCTATTAACCACTCA
human-mt-DLOOP (R)	TTCGCCTGTAATATTGAACGTA
human-mt-18S (F)	TAGA GGGACAAGTGGCGTTC
human-mt-18S (R)	CGCTGAGCCAGTCAGTGT
human-TERT (F)	TCACGGAGACCACGTTTCAAA
human-TERT (R)	TTCAAGTGCTGTCTGATTCCAAT
